# Quinolone Resistance Reversion by Targeting the SOS Response

**DOI:** 10.1128/mBio.00971-17

**Published:** 2017-10-10

**Authors:** E. Recacha, J. Machuca, P. Díaz de Alba, M. Ramos-Güelfo, F. Docobo-Pérez, J. Rodriguez-Beltrán, J. Blázquez, A. Pascual, J. M. Rodríguez-Martínez

**Affiliations:** a Unidad de Enfermedades Infecciosas, Microbiología y Medicina Preventiva, Hospital Universitario Virgen Macarena, Seville, Spain; b Departamento de Microbiología, Universidad de Sevilla, Seville, Spain; c Red Española de Investigación en Patología Infecciosa (REIPI), Instituto de Salud Carlos III, Madrid, Spain; d Instituto de Biomedicina de Sevilla (IBiS), Hospital Universitario Virgen del Rocío/CSIC/Universidad de Sevilla, Seville, Spain; e Centro Nacional de Biotecnología, Consejo Superior de Investigaciones Científicas, Madrid, Spain; f Unidad de Enfermedades Infecciosas, Microbiología y Medicina Preventiva, Hospital Universitario Virgen del Rocio, Seville, Spain; Louis Stokes Veterans Affairs Medical Center

**Keywords:** RecA, SOS response, quinolones, resensitization of antibiotic-resistant bacteria, resistance reversion

## Abstract

Suppression of the SOS response has been postulated as a therapeutic strategy for potentiating antimicrobial agents. We aimed to evaluate the impact of its suppression on reversing resistance using a model of isogenic strains of *Escherichia coli* representing multiple levels of quinolone resistance. *E. coli* mutants exhibiting a spectrum of SOS activity were constructed from isogenic strains carrying quinolone resistance mechanisms with susceptible and resistant phenotypes. Changes in susceptibility were evaluated by static (MICs) and dynamic (killing curves or flow cytometry) methodologies. A peritoneal sepsis murine model was used to evaluate *in vivo* impact. Suppression of the SOS response was capable of resensitizing mutant strains with genes encoding three or four different resistance mechanisms (up to 15-fold reductions in MICs). Killing curve assays showed a clear disadvantage for survival (Δlog_10_ CFU per milliliter [CFU/ml] of 8 log units after 24 h), and the *in vivo* efficacy of ciprofloxacin was significantly enhanced (Δlog_10_ CFU/g of 1.76 log units) in resistant strains with a suppressed SOS response. This effect was evident even after short periods (60 min) of exposure. Suppression of the SOS response reverses antimicrobial resistance across a range of *E. coli* phenotypes from reduced susceptibility to highly resistant, playing a significant role in increasing the *in vivo* efficacy.

## IMPORTANCE

The rapid rise of antibiotic resistance in bacterial pathogens is now considered a major global health crisis. New strategies are needed to block the development of resistance and to extend the life of antibiotics. The SOS response is a promising target for developing therapeutics to reduce the acquisition of antibiotic resistance and enhance the bactericidal activity of antimicrobial agents such as quinolones. Significant questions remain regarding its impact as a strategy for the reversion or resensitization of antibiotic-resistant bacteria. To address this question, we have generated *E. coli* mutants that exhibited a spectrum of SOS activity, ranging from a natural SOS response to a hypoinducible or constitutively suppressed response. We tested the effects of these mutations on quinolone resistance reversion under therapeutic concentrations in a set of isogenic strains carrying different combinations of chromosome- and plasmid-mediated quinolone resistance mechanisms with susceptible, low-level quinolone resistant, resistant, and highly resistant phenotypes. Our comprehensive analysis opens up a new strategy for reversing drug resistance by targeting the SOS response.

## INTRODUCTION

Efforts to overcome the problem of resistance have focused mainly on modifying existing antibiotics by circumventing the molecular mechanisms conferring resistance (1). While such efforts are efficacious against resistant strains, new resistance mechanisms often arise in the process of adaptation to new antimicrobial agents (2, 3).

Members of the *Enterobacteriaceae* family, like *Escherichia coli*, are among the most common causes of community and nosocomial infections. Fluoroquinolones are used for empirical and directed therapy in infections caused by *E. coli* (4). Quinolone resistance has increased notably in *Enterobacteriaceae* from both human and veterinary isolates (5, 6). Mechanisms of fluoroquinolone resistance occur principally through chromosomal mutations in genes encoding the quinolone targets (DNA gyrase and topoisomerase IV), and to a lesser extent through decreased permeability (6). Plasmid-mediated quinolone resistance mechanisms have also been described (7). These determinants on their own (whether chromosomally or plasmid mediated) confer low-level quinolone resistance (LLQR), so that multiple mechanisms must be combined to achieve clinical resistance.

New strategies are needed to block the development of resistance and to extend the life of antibiotics such as quinolones. Multiple studies suggest that adaptive resistance mutations and the acquisition of resistance genes by bacteria are induced or facilitated by antibiotic therapy due to the activation of RecA (leading to the SOS response, the DNA repair and mutagenesis pathway) (8–10). Antibiotics can trigger bacterial stress at both lethal and sublethal concentrations (8). In this respect, fluoroquinolones are potent inducers of the SOS response, causing DNA damage or arresting replication forks by blocking DNA gyrase (10, 11). The SOS pathway is initiated through the activation of RecA, which in turn induces autocatalytic cleavage of the LexA repressor and induces the SOS response genes (8, 10). RecA is involved in DNA repair, recombination, induction of the SOS response, horizontal gene transfer, and biofilm formation (10, 12–14). Systematically altering bacterial SOS activity, both constitutive SOS activation and inactivation, has been revealed as a therapeutic strategy for potentiating bactericidal antibiotics like quinolones against highly susceptible wild-type *E. coli* (15, 16). Several compounds have also been shown to inhibit the ATPase activity of RecA *in vitro* (17–19). Phthalocyanine tetrasulfonate were recently characterized as an *in vivo* RecA inhibitor (20). In short, the SOS response is a promising target for developing therapeutics to enhance the bactericidal activity of antimicrobial agents such as quinolones.

**FIG 1:**
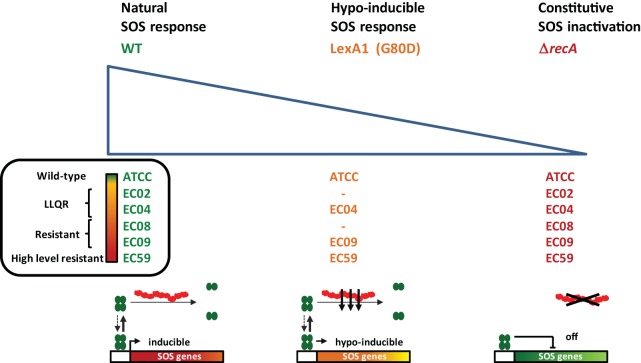
Engineered *recA* and *lexA* variants in *E. coli* displaying a range of SOS activities. The LexA protein is represented by green ovals, and *lexA1* (G80D) cleavage mutations are labeled in orange. RecA is shown as red filaments, and *recA* deleted mutants are labeled in red. Strains with a natural SOS response are labeled in green. Four *lexA1* mutants and six mutants with *recA* deleted were derived from low-level quinolone-resistant (LLQR), resistant, and high-level resistant wild-type (WT) phenotypes of quinolone resistance, allowing the bacterial response to quinolones to be examined across a spectrum of SOS activity. Activated RecA filaments led to cleavage of LexA and inducible expression of SOS genes in the WT strain. Deletion of *recA* (Δ*recA*) inactivated the SOS response. Mutations in the LexA protein (G80D) reduce the rate of self-cleavage relative to the WT strain and so affect the level of SOS induction.

Despite genetic data implicating the SOS response as critical to the survival and adaptation of highly susceptible wild-type bacteria, significant questions remain regarding its impact as a strategy for the reversion or resensitization of antibiotic-resistant bacteria under therapeutic concentrations (21–23). To address this question, we generated *E. coli* mutants that exhibited a spectrum of SOS activity, ranging from a natural SOS response to a hypoinducible (LexA1; very low cleavage rate) (24) or constitutively suppressed response (Δ*recA*) (Fig. 1). We tested the effects of these mutations on a set of isogenic strains carrying different combinations of chromosome- and plasmid-mediated quinolone resistance mechanisms with susceptible, LLQR, resistant, and highly resistant phenotypes. Our comprehensive analysis opens up a new strategy for reversing drug resistance by targeting the SOS response.

(This study was presented in part at ASM Microbe, Boston, MA, in 2016 [oral presentation, session 374].)

## RESULTS

### Suppression of the SOS response sensitizes fluoroquinolone resistance.

Six isogenic *E. coli* strains harboring frequent chromosomal mutations, associated with fluoroquinolone resistance, in the *gyrA* and/or *parC* genes and/or a deletion in the *marR* gene and combined with plasmid-mediated quinolone resistance (PMQR) mechanism (*qnrS*) (ranging from wild-type high level of susceptibility to a high level of resistance) were used for *recA* deletion or *lexA* replacement by *lexA1* and evaluated for susceptibility to quinolones (Table 1; also see Table S1A in the supplemental material).

10.1128/mBio.00971-17.10 TABLE S1 (A) Genotypes and fluoroquinolone susceptibility (by microdilution) of isogenic strains. (B) Oligonucleotides and plasmids used in this study. Download TABLE S1, DOCX file, 0.02 MB. Copyright © 2017 Recacha et al. 2017 Recacha et al. This content is distributed under the terms of the Creative Commons Attribution 4.0 International license.

**TABLE 1:** Genotypes and ciprofloxacin susceptibility (by Etest) of isogenic strains

Strain	Genotype^a^						MIC^b^	CC (CLSI/ EUCAST)^c^	Fold change^d^	Source or reference
	*gyrA1*	*gyrA2*	*parC*	*marR*	*qnr*	SOS system				
ATCC^e^						WT^f^	0.008	S/S		Lab collection
ATCCrecA						Δ*recA*	<0.002	S/S	>4	This study
ATCClexA1						*lexA1*	0.004	S/S	2	This study

EC02	S83L					WT	0.25	S/S		31
EC02recA	S83L					Δ*recA*	0.03	S/S	8	This study

EC04	S83L		S80R			WT	0.5	S/S		31
EC04recA	S83L		S80R			ΔrecA	0.125	S/S	4	This study
EC04lexA1	S83L		S80R			*lexA1*	0.5	S/S	1	This study

EC08	S83L	D87N	S80R			WT	2	**I/R**		31
EC08recA	S83L	D87N	S80R			Δ*recA*	0.5	**S/S**	4	This study

EC09	S83L	D87N	S80R	Δ*marR*		WT	8	**R/R**		31
EC09recA	S83L	D87N	S80R	Δ*marR*		ΔrecA	1	**S/I**	8	This study
EC09lexA1	S83L	D87N	S80R	Δ*marR*		*lexA1*	2	**I/R**	4	This study

EC59	S83L	D87N	S80R	Δ*marR*	*qnrS*	WT	>32	R/R		31
EC59recA	S83L	D87N	S80R	Δ*marR*	*qnrS*	ΔrecA	4	R/R	>8	This study
EC59lexA1	S83L	D87N	S80R	Δ*marR*	*qnrS*	*lexA1*	32	R/R	>1	This study

a Strains are isogenic to *E. coli* ATCC 25922 and carry only the chromosomal modifications, *qnrS* gene, and/or SOS dysfunction (*recA* deletion or nonproteolizable LexA variant [LexA1]). Resistance-associated mutations located in the GyrA and ParC proteins have been defined as resistance mechanisms that alter the target site.

b MIC (in milligrams per liter) of ciprofloxacin by Etest.

c CC, clinical category according to the CLSI and EUCAST breakpoints (25, 38). The clinical category according to the CLSI breakpoint is shown before the slash, and the clinical category according to the EUCAST breakpoint is shown after the slash. The clinical categories are shown as follows: S, susceptible; I, intermediate susceptibility; R, resistant. Subgroups with clinical category changes are indicated in boldface type.

d Fold reduction of MIC compared to the MIC of wild-type strain for the SOS system in each isogenic subgroup.

e *E. coli* ATCC 25922.

f WT, wild-type.

We first confirmed that the Δ*recA* and *lexA1* mutations produce the expected perturbations in the SOS response (significant differences were observed confirming suppression and hypoinduction of the SOS response, respectively) (Fig. S1). The reductions in the MICs of ciprofloxacin ranged from 1-fold to >8-fold against both the Δ*recA* and LexA1 strains (Table 1). Sensitization was greater in Δ*recA* strains (with constitutive SOS inactivation), ranging from 4-fold to >8-fold (Table 1; Fig. S2). Of note, the EC04lexA1 strain did not reduce the MIC values of most the quinolones, which lends support to the hypoinducible SOS response as a less effective strategy of sensitization to quinolones. The process of sensitization was equally efficient across susceptible, LLQR, and resistant phenotypes and independent of the type of molecular mechanism involved in quinolone resistance or whether it was chromosomally or plasmid mediated. Interestingly, *recA* inactivation in the EC02 strain (carrying a GyrA protein with S83L substitution) modified the ciprofloxacin MIC value below the epidemiological cutoff (0.032 mg/liter) (http://www.eucast.org) (25). Here we show that, in terms of MICs, SOS inactivation suppresses the effects of first-step mutations toward resistance associated with topoisomerase type II modifications.

10.1128/mBio.00971-17.2 FIG S1  Monitoring the SOS response in engineered *recA* and *lexA* variants obtained from isogenic quinolone-resistant *E. coli*. Ciprofloxacin (CIP) induces DNA damage leading to activation of the SOS response. Ciprofloxacin-induced green fluorescent protein (GFP) expression from the pMSrecA-gfp vector was used to quantify *recA* promoter activity (SOS induction) after the addition of a fluoroquinolone (ciprofloxacin) at a sublethal concentration (1× MIC, relative to the MIC of the wild-type SOS or not) or fixed concentration (1 mg/liter or 2.5 mg/liter). Data were normalized to the noninduced control (by extracting the fluorescent background) and relative to bacterial density (optical density at 595 nm [OD_595_]). No data are shown for the ATCC 25922 (ATCC) strain at fixed concentrations (1 mg/liter or 2.5 mg/liter) due to the extremely high susceptibility under these conditions. Values that are significantly different are indicated by asterisks as follows: *, *P* < 0.05; **, *P* < 0.01; ***, *P* < 0.001. (Bottom) Representative ciprofloxacin MIC gradient disks for strains EC09 (left) and EC09recA (right) harboring a pMSrecA-gfp vector, and green fluorescent bacteria corresponding to strain EC09 are shown. Download FIG S1, PDF file, 0.5 MB. Copyright © 2017 Recacha et al. 2017 Recacha et al. This content is distributed under the terms of the Creative Commons Attribution 4.0 International license.

10.1128/mBio.00971-17.3 FIG S2  Ciprofloxacin susceptibility testing for three representative isogenic groups of strains (strains EC08, EC09, and EC59) using the MIC gradient strip method. It was observed that constitutive SOS inactivation (*recA* gene deletion) has a clear influence on ciprofloxacin activity, leading to a significant decrease in MIC. MIC values (shown in milligrams per liter) are indicated at the bottom right of each image. Download FIG S2, PDF file, 2 MB. Copyright © 2017 Recacha et al. 2017 Recacha et al. This content is distributed under the terms of the Creative Commons Attribution 4.0 International license.

Similar results were observed for all quinolones tested. The MICs were reduced up to 8-, 8-, 15-, 4-, 4-, and 2-fold for ciprofloxacin, levofloxacin, moxifloxacin, norfloxacin, ofloxacin, and nalidixic acid, respectively (Table S1A).

In addition, the changes in the ciprofloxacin MICs observed for EC08 (MIC of 2 mg/liter down to 0.5 mg/liter) and EC09 (MIC of 8 mg/liter down to 1 mg/liter) *recA* deficient strains involved changes to the susceptible category. Here, strain EC08, which is intermediate or resistant according to the CLSI and EUCAST breakpoints, respectively, was sensitized to susceptible according to both committees. Similarly, the resistant strain, EC09, was sensitized to susceptible and intermediate-susceptible according to the CLSI and EUCAST breakpoints, respectively (Table 1). The clinical category was also changed to susceptible against levofloxacin and moxifloxacin (Table S1A).

These data all lend support to suppression of the SOS response as capable of resensitizing mutant strains with genes encoding three, or even four, different mechanisms of acquired quinolone resistance. The degree of sensitization could be considered moderate (up to 15-fold).

### SOS suppression enhances bactericidal activity against resistant strains.

**FIG 2:**
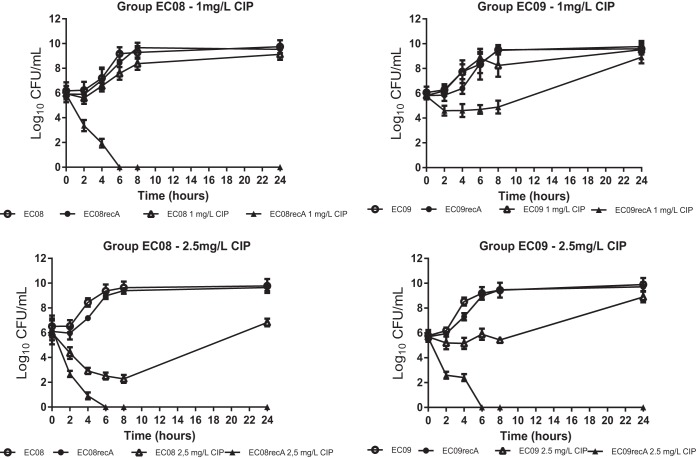
SOS inactivation enhances bactericidal activity against resistant strains. Viable bacterial counts of the EC08/EC08recA and EC09/EC09recA isogenic pairs in time-kill assays at ciprofloxacin (CIP) concentrations of 1 mg/liter (breakpoint for resistance according to EUCAST) and 2.5 mg/liter (human serum *C*_max_), respectively. Data are represented as the means from at least three independent measurements. Standard deviations are indicated by the error bars (standard deviations not shown are smaller than the symbols).

To show the impact of SOS response suppression in terms of bacterial viability, time-kill curves were obtained for each isogenic group according to the SOS system induction status. At fixed concentrations, a marked reduction of viable bacteria was observed with the inactivated SOS response over 24 h of incubation (Fig. 2). At 1 mg/liter, a bactericidal effect (drop of >3 log_10 _CFU/ml) was observed against strain EC08recA after 4 h (and no viable bacteria were recovered after 6 h). At 2.5 mg/liter, a bactericidal effect was observed against both EC08 (after 4 h) and EC08recA (after 2 h), although regrowth was observed after 24 h in strain EC08 (6.8 log_10_ CFU/ml) but not strain EC08recA (Fig. 2). At 1 mg/liter, a bacteriostatic effect only (drop of <3 log_10 _CFU/ml) was observed in strain EC09recA (although a marked difference was observed in the first 8 h compared to EC09, up to Δ4.2 log_10_ CFU/ml at 6 h). At 2.5 mg/liter, however, a bactericidal effect was observed in EC09recA (after 2 h) and a bacteriostatic effect in EC09 in the first 8 h, with regrowth after 24 h (Fig. 2). Under these conditions, SOS induction suppression leads to a high bactericidal effect under relevant therapeutic concentrations in *E. coli* harboring multiple mechanisms of quinolone resistance.

At 1× MIC of ciprofloxacin (of the tested strains harboring a nonmodified SOS system), major differences in the numbers of viable bacteria were observed after 24 h (Δ6.1, Δ4.2, Δ9.5, and Δ6.9 log_10_  CFU/ml in the ATCC, EC04, EC08, and EC09 isogenic pairs, respectively). Minor differences were observed at 4× MIC of ciprofloxacin (Fig. S3). Additional kinetic assays (growth curves and ATP production) confirmed these results (Fig. S8).

10.1128/mBio.00971-17.4 FIG S3  SOS inactivation enhances bactericidal activity against resistant strains. Viable bacterial counts of ATCC 25922 (ATCC)/ATCCrecA, EC04/EC04recA, EC08/EC08recA, and EC09/EC09recA isogenic pairs in time-kill assays with ciprofloxacin (CIP) concentrations of 1× MIC and 4× MIC (relative to the MICs of the wild-type SOS), respectively. The data are represented as the means from at least three independent measurements. Standard deviations are indicated by the error bars (standard deviations not shown are smaller than the symbols). Download FIG S3, PDF file, 0.4 MB. Copyright © 2017 Recacha et al. 2017 Recacha et al. This content is distributed under the terms of the Creative Commons Attribution 4.0 International license.

### Suppression of SOS response reduces survival in resistant strains after a short time.

**FIG 3:**
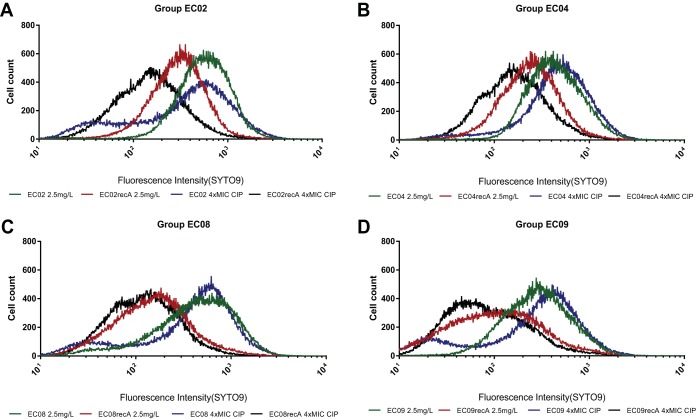
Flow cytometry of four groups of isogenic LLQR cells (harboring a natural [wild-type] or inactive SOS system [Δ*recA*]) treated for 60 min at 2.5 mg/liter or at 4× MIC (relative to the MIC of the wild-type SOS) of ciprofloxacin. Survival was measured as staining with green fluorescent SYTO9.Strains EC02 (A), EC04 (B), EC08 (C), and EC09 (D) were studied.

The LIVE/DEAD staining method was tested using three different approaches to show the impact of SOS inactivation on bacterial survival during a short period of exposure to quinolones. First, using the Infinite 200PRO multireader, it was clear that the live/dead ratio depended significantly on the SOS response functionality. Figure S4A shows the quantitative results obtained at 1× MIC of ciprofloxacin (according to the MICs of strains with a functional SOS response) after 4 h of exposure. Under these conditions, the ratio of live to dead cells for *E*. *coli* ATCCrecA, EC04recA, EC08recA, and EC09recA deficient SOS variants decreased by 88%, 94%, 98%, and 98%, respectively, compared to their parental variants with the wild-type SOS response (*P* < 0.001). This reduction was also proportional to the level of quinolone resistance (*P* < 0.001). Second, these results were supported by fluorescence microscopy assay. Figure S4A shows representative images of strains EC08 and EC08recA exposed to 2.5 mg/liter (maximum concentration of drug in serum [*C*_max_]) of ciprofloxacin for 4 h, supporting the differential response. Third, in order to determine whether the SOS response was a key factor for survival after a very short period of exposure to bactericidal drugs like quinolones in strains with mechanisms of acquired resistance (strains EC02, EC04, EC08, and EC09), flow cytometry was used to examine bacterial viability after 60 min of exposure at multiple concentrations of ciprofloxacin (see Materials and Methods). A significant reduction in cell viability was observed following treatment with ciprofloxacin at 4× MIC and 2.5 mg/liter (Fig. 3 and S4B) (also at 1× MIC and 1 mg/liter [data not shown]), which correlates directly with the inability to activate the SOS response. These results imply that the SOS response is a key short-term responder to DNA damage in both LLQR and resistant *E. coli* at clinically relevant quinolone concentrations.

10.1128/mBio.00971-17.5 FIG S4  LIVE/DEAD staining. (A) Quantification of isogenic resistant *E. coli* strains exposed to 1× MIC of ciprofloxacin (relative to the MICs of strains with a functional SOS system) for 4 h. Representative images of strains EC08/EC08recA exposed to 2.5 mg/liter (*C*_max_) of ciprofloxacin for 4 h are shown. Significant *P* values are noted (*, *P* <0.001). (B) Flow cytometry of the EC08 group of isogenic LLQR cells (harboring a natural [WT] or inactive SOS system [Δ*recA*]) treated for 60 min at 2.5 mg/liter or at 4× MIC (relative to the MIC of the strain with wild-type SOS) of ciprofloxacin. Survival was measured as staining with red fluorescent propidium iodine (PI). Download FIG S4, PDF file, 0.6 MB. Copyright © 2017 Recacha et al. 2017 Recacha et al. This content is distributed under the terms of the Creative Commons Attribution 4.0 International license.

### Pharmacokinetics and pharmacodynamics of the *in vivo* model.

The fit of the mathematical model to the mouse serum data (i.e., correlation between the observed versus predicted data of the mathematical model) was acceptable (*R*^2^ = 0.968). The estimated parameter values were as follows: clearance (CL), 0.38 × 10^−1^ liters/h; volume of distribution of the drug in the peripheral compartment (*V*_p_) of 0.95 × 10^−2^ liters; transfer rate constant from central to peripheral compartment (*k*_cp_) of 4.03; transfer rate constant from peripheral to central compartment (*k*_pc_) = 0.44 ×10^−7^ (Fig. S5). AUC/MIC values of 12.2/24.4 for strain EC08 and 48.8/97.6 for strain EC08recA were predicted in our model for 50 and 100 mg/kg of body weight, respectively.

10.1128/mBio.00971-17.6 FIG S5  Pharmacokinetic conditions for *in vivo* murine model. Download FIG S5, PDF file, 0.1 MB. Copyright © 2017 Recacha et al. 2017 Recacha et al. This content is distributed under the terms of the Creative Commons Attribution 4.0 International license.

### SOS suppression enhances bactericidal activity against resistant strains *in vivo.*

**FIG 4:**
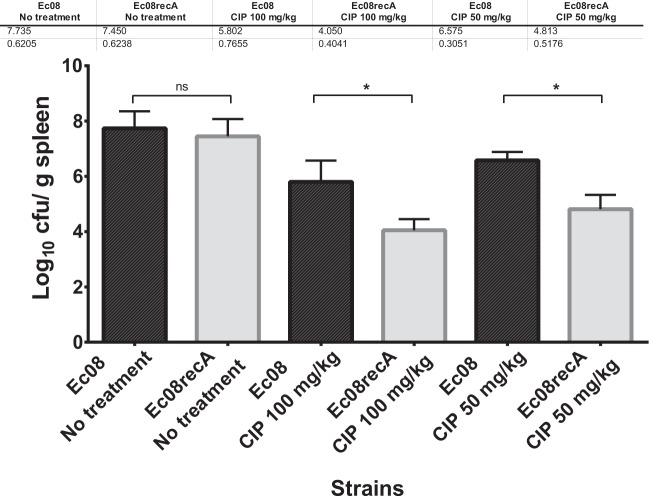
SOS inactivation reduces the *in vivo* survival of mice infected with bacteria receiving quinolone treatment. Efficacies of ciprofloxacin (CIP) in a murine model of sepsis caused by strains EC08 (intact SOS system) and EC08recA (inactivated SOS system). Group 1 was given ciprofloxacin 50 mg/kg q12h intraperitoneally. Group 2 was given ciprofloxacin 100 mg/kg q12h intraperitoneally. The control group was not treated with ciprofloxacin. Standard deviations are indicated by the error bars. Values that were significantly different (*P* < 0.001) are indicated by a bar and asterisk. Values that were not significantly different (ns) are indicated.

Selected isogenic mutants, ciprofloxacin-nonsusceptible strain EC08 and susceptible strain EC08ΔrecA, were included in a murine model of intraperitoneal sepsis. No differences of bacterial load were observed in the spleens of control groups infected with strains EC08 and EC08ΔrecA (7.73 ± 0.62 versus 7.45 ± 0.61 log_10_ CFU/g). All the controls died in the first 48 h according to the minimum lethal dose (MLD), with no differences between the strains (*P* > 0.05). Note that 33% mortality was observed within the first 24 h in the EC08 group treated with 50 mg/kg every 12 h (q12h), while no mortality was observed in the remaining treated groups during the experiments. With respect to bacterial burden, in mice infected with strain EC08ΔrecA (with the inactivated SOS response), treatment with ciprofloxacin at 50 mg/kg q12h and at 100 mg/kg q12h significantly reduced bacterial concentrations (Δlog_10_ CFU/g units of 1.75 and 1.76 in the spleen; *P* < 0.001, respectively) with respect to groups infected with strain EC08 (with the active SOS response) (Fig. 4).

## DISCUSSION

The SOS response plays an important role in adaptation and acquired bacterial resistance to antibiotics. The key regulators (LexA and RecA) have been proposed as an attractive strategy for increasing bacterial sensitivity to antibiotics and combating the emergence of resistance. This strategy has been tested essentially against highly susceptible wild-type bacteria without molecular mechanisms of acquired resistance (16, 26–28). Low-level resistance phenotypes, such as LLQR (which can be exposed to sublethal levels of antibiotics during antimicrobial treatment), pose a significant threat to the development of clinical resistance (29–31). Previous data validating the SOS response as a target of interest motivated our efforts to explore the consequences of a broader spectrum of SOS activity, ranging from natural through hypoinducible to constitutively repressed SOS response (Fig. 1) in a set of isogenic strains carrying combinations of chromosome- and plasmid-mediated quinolone resistance, and phenotypes ranging from susceptible to LLQR, resistant, and highly resistant. Our detailed analysis opens up a new strategy for reversing drug resistance by targeting the SOS response.

The bactericidal activity of quinolones in bacteria has been related to a combination of DNA fragmentation, reactive oxygen species (ROS) production, and programmed cell death systems, such as *mazEF* (32–35). The SOS response has also been postulated as a formidable strategy against aggressions such as antimicrobial exposure (10). The link between quinolones, activation of the SOS response, and induction of antibiotic resistance (26, 28) demonstrates the potential for reducing resistance by targeting the RecA and LexA proteins that are essential for an SOS response. Our study provides evidence that suppression of the SOS pathway can synergize with specific antimicrobial agents, such as quinolones, to reduce MICs in a process of resistance reversion. In the case of constitutive SOS inactivation, the MIC data of Δ*recA* mutants were in agreement with earlier studies of highly susceptible wild-type phenotypes (36, 37), and resensitization was observed in LLQR, resistant, and highly resistant phenotypes (Table 1 and Fig. 2 and 4; also see Fig. S6 in the supplemental material). However, the increased sensitivity was less when the level of SOS induction was attenuated by a slow-cleaving LexA variant, the LexA1 (G80D) strain, which showed minor changes in MIC in both susceptible and resistant phenotypes. This discrepancy could be due, in part, because *recA* deletion can have an impact beyond leading to loss of LexA cleavage and SOS response suppression, with additional implications in important processes like homologous recombination. An overactive SOS response can also increase quinolone susceptibility, although to a lesser extent than constitutive inhibition (15). Moreover, several compounds that inhibit RecA *in vitro* or *in vivo* have been discovered (17–20). In short, potent inhibition of the SOS response in concert with DNA-damaging agents like quinolones offers the best option for potential synergy, and we focused our study on *recA* mutants in order to show their impact on the reversion of quinolone resistance.

10.1128/mBio.00971-17.7 FIG S6  Impact of the SOS response on cell growth in the presence of quinolones. Growth curve data, at 8 h and 24 h, of strains exposed to sublethal stress at 0.5× MIC relative to the MIC of a strain with wild-type SOS (A) and to a 1 mg/liter fixed concentration (B) and a 2.5 mg/liter fixed concentration of ciprofloxacin stress (C). The data are represented as the means from five independent measurements. Significant *P* values are noted (*, *P* < 0.01; **, *P* < 0.001). Download FIG S6, PDF file, 0.4 MB. Copyright © 2017 Recacha et al. 2017 Recacha et al. This content is distributed under the terms of the Creative Commons Attribution 4.0 International license.

**FIG 5:**
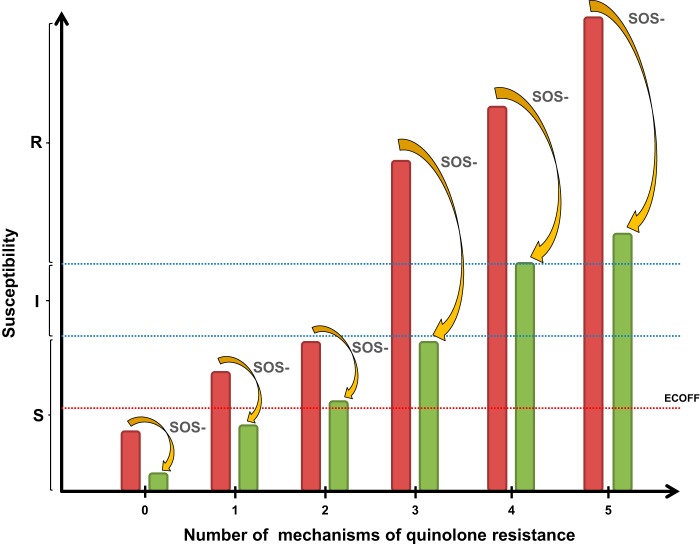
A general qualitative model illustrating the efficacy of SOS response suppression for reversion of fluoroquinolone resistance. Reversion of resistance was even observed in bacteria carrying genes encoding four different resistance mechanisms. Red bars correspond to *E. coli* with an intact SOS system; green bars correspond to *E. coli* with a suppressed SOS response. The epidemiological cutoff (ECOFF) (0.032 mg/liter [http://www.eucast.org]) is indicated by the red dotted line. S, I, and R mean susceptibility, intermediate susceptibility, and clinical resistance, respectively. SOS- means SOS response suppression.

According to the CLSI guidelines (38), complete inactivation of the SOS response led to a change in clinical category for ciprofloxacin from intermediate or resistant to susceptible in EC08 (S83L, D87N, and S80R substitutions) and EC09 (S83L, D87N, and S80R substitutions and Δ*marR*) strains, respectively. Using EUCAST guidelines (25), inactivation changed the clinical category from resistant to susceptible in strain EC08 and to intermediate-susceptible in strain EC09, respectively. These results support the relevance of a strategy of SOS inactivation for bringing about reversion of antimicrobial resistance at a level that could be clinically significant. Interestingly, the inactivation of *recA* in the EC02 strain (encoding an S83L substitution) modified the ciprofloxacin MIC below the epidemiological cutoff (0.032 mg/liter; http://www.eucast.org) (39, 40). Here we show that inactivation of the SOS system suppresses the effect, in terms of MIC, of the first step toward resistance associated with topoisomerase type II modifications. A qualitative model illustrating the efficacy of SOS suppression in the resensitization of quinolone resistance is shown in Fig. 5, showing that this phenomenon is observed in bacteria with genes encoding multiple (up to four) different resistance mechanisms.

In terms of kinetic assays, multiple approaches were developed to evaluate the reversion of quinolone resistance mediated by an inactivated SOS response at both long and short periods of exposure to drugs. In all cases, we observed a clear selective disadvantage for survival in strains with a suppressed SOS response when exposed to ciprofloxacin at relevant concentrations (breakpoint concentrations, serum *C*_max_, and MIC values) (Fig. 2 and 3 and S6). For time-kill curves, an inactivated SOS response in *E. coli* harboring multiple mechanisms of resistance had a high bactericidal effect in the presence of clinically relevant ciprofloxacin concentrations after 2 to 4 h of exposure (depending on the strain and conditions), which was maintained for 24 h (Fig. 2 and S3). Flow cytometry assays also showed significant reductions in cell viability following a short period of exposure to the drug (60 min), which was directly related to the inability to activate the SOS response (Fig. 3). Our data show that changes in the MICs of specific quinolone-resistant strains (EC08 and EC09) as a result of an inactivated SOS response correlated with ROS formation at clinically relevant concentrations of ciprofloxacin (Fig. S7). In terms of bacterial viability, these data support the potential utility of this strategy for resensitizing or reversing quinolone resistance after both short and long periods of exposure to quinolones at relevant concentrations. Interestingly, whether SOS response suppression could restrict the evolution to clinical resistance in LLQR phenotypes should be tested (16).

10.1128/mBio.00971-17.8 FIG S7  SOS inactivation increases ROS production in resistant isogenic *E. coli* strains at clinically relevant ciprofloxacin (CIP) concentrations. Bacterial cells were treated with ciprofloxacin at a concentration of 1 mg/liter. Significant *P* values are noted (*, *P* < 0.05; ** *P* < 0.001). ns means nonsignificant differences. Download FIG S7, PDF file, 0.3 MB. Copyright © 2017 Recacha et al. 2017 Recacha et al. This content is distributed under the terms of the Creative Commons Attribution 4.0 International license.

10.1128/mBio.00971-17.9 FIG S8  Impact of SOS response on cell growth and ATP production in the presence of quinolones. (A) Growth curves of strains exposed to sublethal stress at 0.5× MIC, relative to the MIC of the wild-type SOS phenotype, and at fixed 1 mg/liter and 2.5 mg/liter concentrations of ciprofloxacin (CIP) stress. (B and C) Monitoring of ATP production of ATCC/ATCCrecA, EC02/EC02recA EC04/EC04recA, EC08/EC08recA, EC09/EC09recA and EC59/EC59recA isogenic pairs in the presence of (B) 2.5 mg/liter concentrations of ciprofloxacin (human serum *C*_max_) or (C) a sublethal concentration of ciprofloxacin at 0.5× MIC, relative to the MIC of the wild-type SOS. Data are shown as the means of at least 5 independent measurements. Standard deviations are indicated by the error bars (standard deviations not shown are smaller than the symbols). Download FIG S8, PDF file, 1.1 MB. Copyright © 2017 Recacha et al. 2017 Recacha et al. This content is distributed under the terms of the Creative Commons Attribution 4.0 International license.

Although SOS response inactivation led to moderate reductions in the MICs of ciprofloxacin (up to 8-fold) and other fluoroquinolones (up to 15-fold), these differences could play a significant role in therapeutic failure, bearing in mind the concentration-dependent character of these antimicrobials, whose predictors of efficacy *in vivo* are *C*_max_/MIC and AUC/MIC. AUC/MIC values of >30 are associated with low mortality and are required for clinical efficacy (41–44). Our murine sepsis model, using isogenic strains that were resistant (EC08) and susceptible (EC08recA; lacking SOS response) to ciprofloxacin according to EUCAST, showed the impact of the pathway on the *in vivo* efficacy of this fluoroquinolone (with a reduction in bacterial count of around 99%). Our murine model shows that inactivation of the SOS pathway in an initially quinolone-resistant *E. coli* strain (EC08) significantly increases the *in vivo* efficacy of ciprofloxacin. According to our data, engineered bacteriophage targeting SOS response (by overexpression of an inactivated LexA variant) was shown to be a promising resistance reversion strategy (45).

In overall terms, this study shows that suppression of the SOS response enhances the bactericidal activity of antimicrobials like quinolones across a range of *E. coli* phenotypes from highly susceptible to highly resistant and plays a significant role in increasing the *in vivo* efficacy of these bactericidal drugs against bacteria with multiple mechanisms of acquired resistance. The development of RecA inhibitors could function as an adjuvant therapy, potentiating antimicrobial activity and contributing to the resensitization or reversion of drug resistance.

## MATERIALS AND METHODS

### Strains, growth conditions, and antimicrobial agents.

Wild-type *E. coli* ATCC 25922 was used as the starting strain for all constructions (Table 1). *E. coli* ATCC 25922 (wild-type) and isogenic EC02, EC04, EC08, EC09, and EC59 strains represent progressive degrees of fluoroquinolone resistance, ranging from susceptible to resistant (see Text S1 in the supplemental material for details).

10.1128/mBio.00971-17.1 TEXT S1  Supplemental Materials and Methods and Results. Download TEXT S1, DOCX file, 0.04 MB. Copyright © 2017 Recacha et al. 2017 Recacha et al. This content is distributed under the terms of the Creative Commons Attribution 4.0 International license.

Liquid or solid Luria-Bertani medium (LB), Mueller-Hinton broth (MHB), and M9 minimal medium were used. Strains were grown at 37°C. The following quinolones were used for the different assays: nalidixic acid, ciprofloxacin, levofloxacin, moxifloxacin, norfloxacin, and ofloxacin (Sigma-Aldrich, Madrid, Spain).

### Isogenic strain construction.

*lexA1* mutants (coding for a LexA G80D substitution) (46) were obtained by gene replacement, as previously described (Table 1 and see Table S1B in the supplemental material) (31, 47). Disruption of the *recA* gene was carried out with a modified version of the method described by Datsenko and Wanner (48). The *qnrS* gene was cloned into the pBK-CMV vector as described previously (31) (see Text S1 for details).

### MICs.

MICs were determined in triplicate for each bacterial strain, using two different techniques, broth microdilution and the Etest technique, and following CLSI reference methods (38). Clinical categories were established according to CLSI and EUCAST breakpoints (25, 38).

### Time-kill curve assays.

To show the effect of suppression of the SOS response on bacterial viability, time-kill assays were performed with each isogenic group based on the SOS system induction status. Mueller-Hinton broth was used with 1× MIC and 4× MIC concentrations of ciprofloxacin. Ciprofloxacin concentrations were relative to MICs for strains harboring the unmodified SOS system (i.e., with intact *recA* and *lexA* genes). Selected isogenic groups of strains, the EC08 and EC09 groups (bordering on clinical resistance) were also exposed to fixed concentrations of antimicrobial (1 mg/liter, the breakpoint for resistance according to EUCAST, or 2.5 mg/liter, human serum *C*_max_ for ciprofloxacin) in MHB (25, 49). Growth in drug-free broth was evaluated in parallel as a control. Cultures were incubated at 37°C with shaking at 250 rpm. An initial inoculum of 10^6^ CFU/ml was used in all experiments, and bacterial concentrations were determined at 0, 2, 4, 6, 8, and 24 h by colony counting.

### Quantification of live/dead bacteria by flow cytometry.

The Molecular Probes LIVE/DEAD BacLight bacterial viability kit (Invitrogen) was used to show the impact of SOS inactivation after a short period of antimicrobial exposure by flow cytometry (Cytomics FC500-MPL; Beckman Coulter) according to the kit instructions.

Cells were exposed at 1× MIC and 4× MIC ciprofloxacin concentration of the tested strains harboring a nonmodified SOS system (i.e., intact *recA* and *lexA* genes) or to a fixed concentration (1 mg/liter, the breakpoint for resistance according to EUCAST, or 2.5 mg/liter, the serum *C*_max_ for ciprofloxacin) (25, 49).

Cells were cultured in the same way and exposed to ciprofloxacin for 60 min. To prepare the cells for measurement, 1 ml of cell culture was washed once in ice-cold phosphate-buffered saline (PBS), resuspended in 1 ml of saline solution, stained according to the kit instructions, and then incubated for 15 min before counting. The following photomultiplier tube (PMT) voltages were used: 420 V for FL1 and 560 V for FL3. At least 10,000 cells per sample were collected. Flow cytometry acquisition was performed at a low flow rate (~30 events/s) (35).

### Mice.

Male immunocompetent C57BL/6 mice were obtained from the University of Seville. The project was approved by the Ethics and Clinical Research Committee of the Virgen Macarena and Virgen del Rocio University Hospitals (reference number 1086-N-15) (see Text S1 for details).

### Pharmacokinetics and pharmacodynamics.

Pharmacokinetic serum data from our previous work were fitted to a two-compartment model (intraperitoneal space and blood) using ADAPT 5 (50, 51). A range of dosages were simulated in order to obtain a favorable pharmacokinetic parameter of area under the concentration-time curve from 0 to 24 h (AUC_0−24_)/MIC ~50 or ~100, adjusted to the SOS-deficient strain in the isogenic pair EC08/EC08recA (strain EC08recA has a ciprofloxacin MIC of 0.5 mg/liter).

### Experimental model.

Mice weighing 16 to 18 g were used. Using a murine model of peritoneal sepsis, the minimum lethal dose (MLD) for EC08 and EC08recA strains was determined (see Text S1 for details). The murine model was used to evaluate the efficacy of ciprofloxacin between strains EC08 and EC08recA. Mice were infected intraperitoneally using the MLD. Two hours postinfection, antimicrobial therapy started. Animals were randomly assigned to different therapeutic groups as follows: group 1,ciprofloxacin administered intraperitoneally at 50 mg/kg of body weight every 12 h (q12h); group 2, ciprofloxacin administered intraperitoneally at 100 mg/kg q12h;control group, no ciprofloxacin treatment. At 24 h, the bacterial loads in the spleens of 15 mice per strain and ciprofloxacin dosage were determined (see Text S1 for details).

### Statistical analysis.

For statistical evaluation, the Student’s *t* test was used when two groups were compared. The analysis of variance (ANOVA) test and Tukey’s posthoc tests were used for group comparisons. Differences were considered significant when *P* values were ≤0.05.
