# Supplementary material for: Quinolone Resistance Reversion by Targeting the SOS Response
Source: mBio. 2017 Oct 10;8(5):e00971-17. doi: 10.1128/mBio.00971-17 (PMC5635686; doi:10.1128/mBio.00971-17)
Supplement: TABLE S1 [file mbo005173521st1.docx]

**Table S1A.** Genotypes and fluoroquinolone susceptibility (by microdilution) of isogenic strains.

| **Strain^a^** | **Mechanism of quinolone resistance** | | | | | **SOS system** | **MIC (Fold)^b,d^** | | | | | | **CC**  **(CLSI/EUCAST)^c^** | **Source or reference** |
| --- | --- | --- | --- | --- | --- | --- | --- | --- | --- | --- | --- | --- | --- | --- |
|  | ***gyrA*1** | ***gyrA*2** | ***parC*** | ***marR*** | ***qnr*** |  | **CIP** | **LVX** | **MXF** | **NFX** | **OFX** | **NAL** |  |  |
| ATCC^e^ | - | - | - | - | - | WT^f^ | 0.004 | 0.015 | 0.015 | 0.03 | 0.03 | 2 | S/S | (30) |
| ATCCrecA | - | - | - | - | - | Δ*recA* | 0.001 (4) | 0.002 (8) | 0.001 (15) | 0.015 (2) | 0.008 (4) | 1 (2) | S/S | This study |
| ATCClexA1 | - | - | - | - | - | *lexA1* | 0.002 (2) | 0.008 (2) | 0.015 (1) | 0.015 (2) | 0.03 (1) | 2 (1) | S/S | This study |
| EC02 | S83L | - | - | - | - | WT | 0.06 | 0.125 | 0.06 | 0.5 | 0.5 | 256 | S/S | (30) |
| EC02recA | S83L | - | - | - | - | Δ*recA* | 0.008 (8) | 0.03 (4) | 0.015 (4) | 0.25 (2) | 0.125 (4) | 128 (2) | S/S | This study |
| EC04 | S83L | - | S80R | - | - | WT | 0.5 | 0.5 | 0.5 | 1 | 0.5 | 256 | S/S | (30) |
| EC04recA | S83L | - | S80R | - | - | Δ*recA* | 0.125 (4) | 0.125 (4) | 0.125 (4) | 0.5 (2) | 0.25 (2) | 256 (1) | S/S | This study |
| EC04lexA1 | S83L | - | S80R | - | - | *lexA1* | 0.5 (1) | 0.5 (1) | 0.5 (1) | 0.5 (2) | 0.5 (1) | 256 (1) | S/S | This study |
| EC08 | S83L | D87N | S80R | - | - | WT | 2 | 2 | 2 | 4 | 4 | 256 | I/R | (30) |
| EC08recA | S83L | D87N | S80R | - | - | Δ*recA* | 0.5 (4) | 0.5 (4) | 0.5 (4) | 1 (4) | 1 (4) | 256 (1) | S/S | This study |
| EC09 | S83L | D87N | S80R | ΔmarR | - | WT | 4 | 4 | 4 | 8 | 8 | 512 | R/R | (30) |
| EC09recA | S83L | D87N | S80R | ΔmarR | - | Δ*recA* | 1 (4) | 1 (4) | 1 (4) | 2 (4) | 2 (4) | 256 (2) | S/I | This study |
| EC09lexA1 | S83L | D87N | S80R | ΔmarR | - | *lexA1* | 2 (2) | 2 (2) | 2 (2) | 2 (4) | 2 (4) | 256 (2) | I/R | This study |
| EC59 | S83L | D87N | S80R | ΔmarR | *qnrS* | WT | 32 | 32 | 32 | 32 | 32 | 512 | R/R | (30) |
| EC59recA | S83L | D87N | S80R | ΔmarR | *qnrS* | Δ*recA* | 8 (4) | 16 (2) | 8 (4) | 8 (4) | 16 (2) | 512 (1) | R/R | This study |
| EC59lexA1 | S83L | D87N | S80R | ΔmarR | *qnrS* | *lexA1* | 32 (1) | 32 (1) | 32 (1) | 32 (1) | 32 (1) | 512 (1) | R/R | This study |

^a^Genotype. Strains are isogenic to *E. coli* ATCC 25922 and carry only chromosomal modifications, *qnrS* gene and/or SOS dysfunction [*recA* deletion or non-proteolizable LexA variants (LexA1)]. Resistance-associated mutations located in the GyrA and ParC proteins have been defined as resistance mechanisms that alter the target site.

^b^MIC (mg/L) of ciprofloxacin by Etest.

^c^ CC (CLSI/EUCAST) means clinical categories for ciprofloxacin according to CLSI or EUCAST breakpoints (24,37).

^d^ Number of fold reduction in MIC of each quinolone compared to wild-type strain for the SOS system in each isogenic subgroup.

^e^ *E. coli* ATCC 25922

^f^ Wild-type

S, susceptible; I, intermediate susceptibility; R, resistance

**Table S1B.** Oligonucleotides and plasmids used in this study.

| **Primer or plasmid** | **Sequence^a^** | **Use in this study** | **Source or reference** |
| --- | --- | --- | --- |
| Gene Replacement | | | |
| Pre-lexA1-Bam | 5´-cgggatcccg GTGATGCTGGTGATGATTGTGGA-3´ | Mutagenesis of *lexA* | This study |
| Post-lexA1-Bam | 5´-cgggatcccg GTAAGGCGAGATGCCAGAGTGCT-3´ | Mutagenesis of *lexA* | This study |
| Pre-lexA1 | 5´-GCGGCATTCCGTCAGGAAGGCGTA-3´ | *lexA* amplification | This study |
| Post-lexA1 | 5´-GCACCAACCGCCACGCCGCCCAA-3´ | *lexA* amplification | This study |
| ST76-1 | 5´-CGACTCACTATAGGGAGACCG-3´ | pST76C cloning testing | (45) |
| ST76-2 | 5´-TGCCTGCAGGTCGACTCTAGAG-3´ | pST76C cloning testing | (45) |
| Inactivation of chromosomal genes | | | |
| H1-recA-P1 | 5´-CAGAACATATTGACTATCCGGTATTACCCGGCATGACAGGAGTAAAAATGGCTATCGACGAAAACAAACA-GTGTAGGCTGGAGCTGCTTC-3´ | *recA* inactivation | This study |
| H2-recA-P2 | 5´-ATGCGACCCTTGTGTATCAAACAAGACGATTAAAAATCTTCGTTAGTTTCTGCTACGCCTTCGCTATCAT-ATGGGAATTAGCCATGGTCC-3´ | *recA* inactivation | This study |
| Pre-H1-recA | 5’ -TCGTCAGGCTACTGCGTATGCAT-3’ | *recA* inactivation testing | This study |
| Post-H2-recA | 5’ -GTACCGCACGATCCAACAGGCGA-3’ | *recA* inactivation testing | This study |
| K1 | 5´ -CAGTCATAGCCGAATAGCCT-3´ | Chromosomal inactivation | (46) |
| K2 | 5´ -CGGTGCCCTGAATGAACTGC-3´ | Chromosomal inactivation | (46) |
| Kt | 5´-CGGCCACAGTCGATGAATCC-3´ | Chromosomal inactivation | (46) |
| **Plasmids** |  |  |  |
| pBK-CMV |  | Cloning vector | Stratagene |
| pST76C |  | Gene replacement/  suicide vector | (45) |
| pUC19RP12 |  | Gene replacement/ resolution vector | (45) |
| pKD4 |  | Chromosomal inactivation | (46) |
| pKOBEG |  | Chromosomal inactivation/ helper vector | (46) |
| pCP20 |  | Chromosomal inactivation/ resolution vector | (46) |
| pMS201 |  | low copy, GFP reporter vector (rep-pSC101 Km^R^ gfp) |  |
| pMSrecA-gfp |  | recA-gfp reporter promoter fusion | ^6^ |

^a^Underlined nucleotides correspond to the *Bam*HI site used for cloning.
