# Supplementary figures and images for: Quinolone Resistance Reversion by Targeting the SOS Response
Source: mBio. 2017 Oct 10;8(5):e00971-17. doi: 10.1128/mBio.00971-17 (PMC5635686; doi:10.1128/mBio.00971-17)

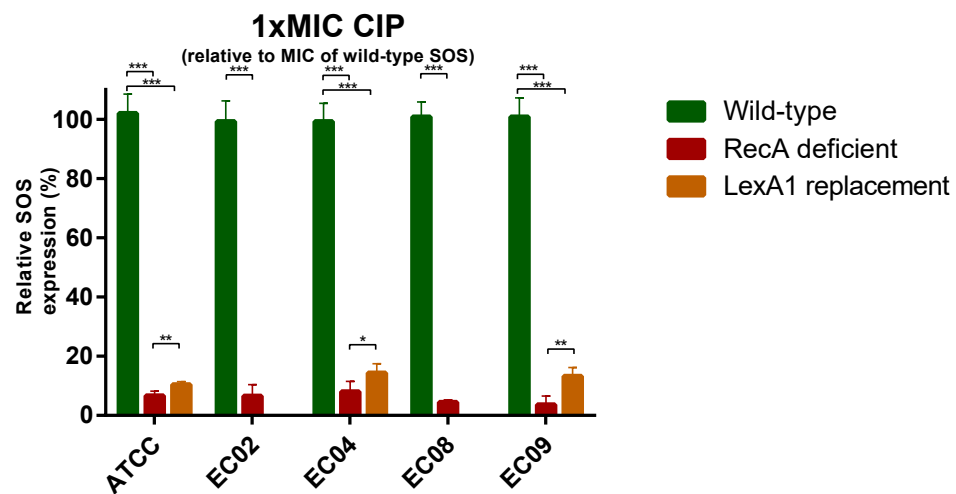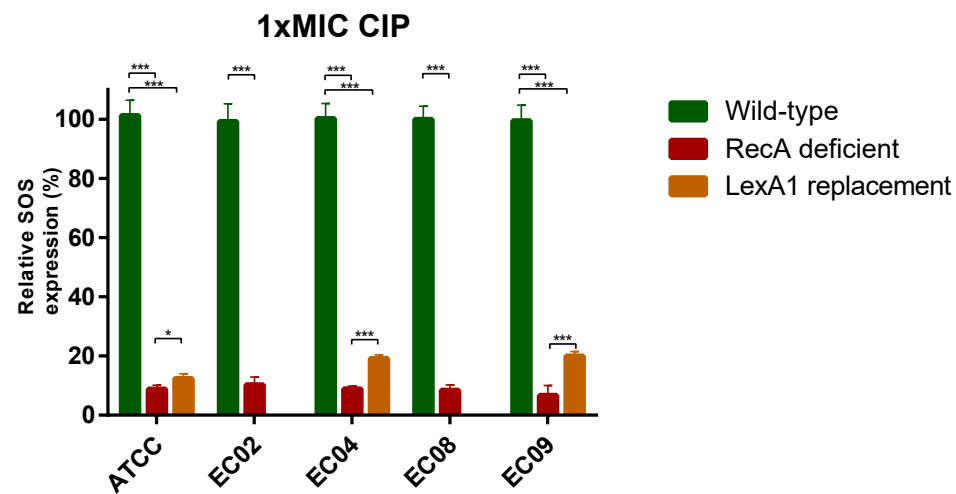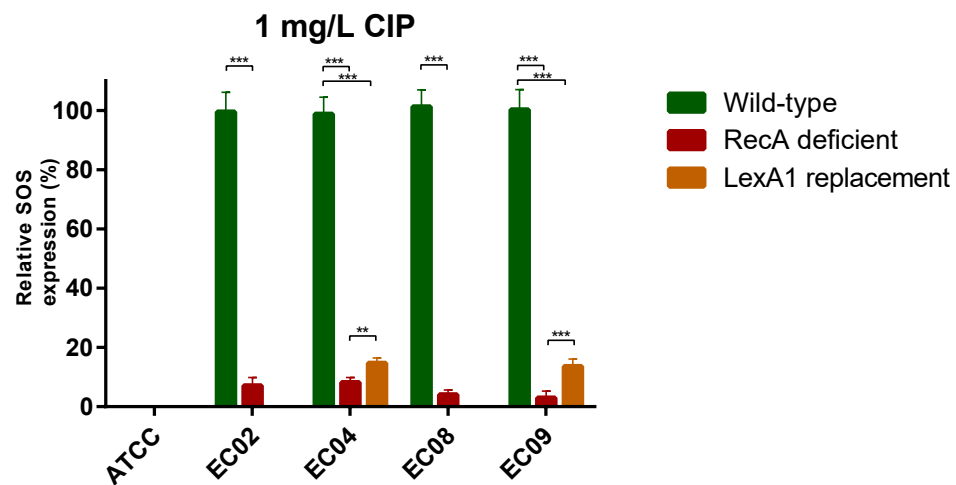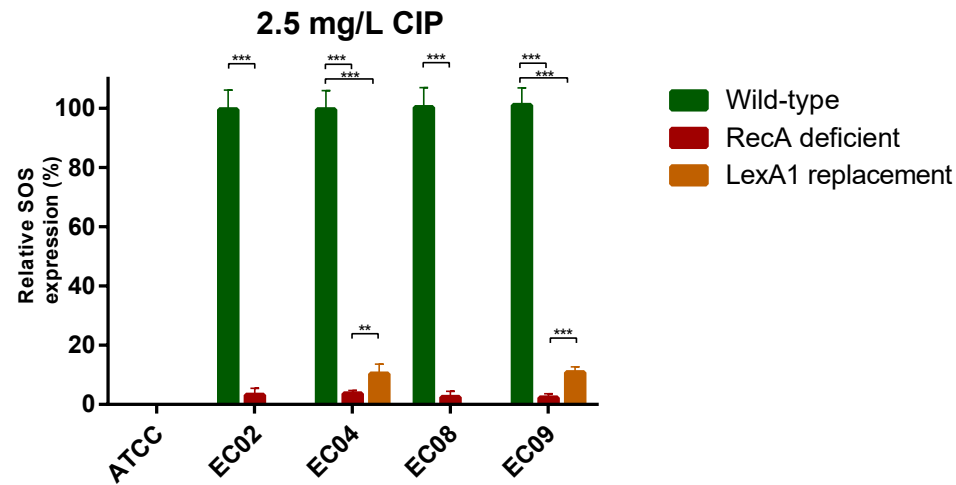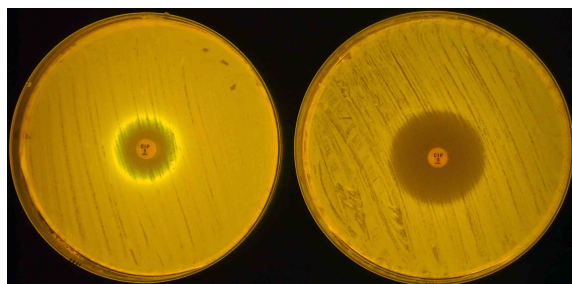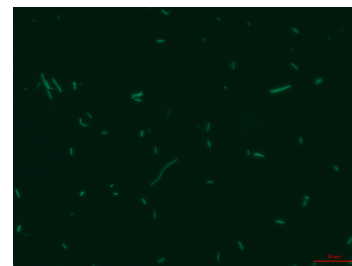

Supplement: FIG S1 [file mbo005173521sf1.pdf]

Figure S2

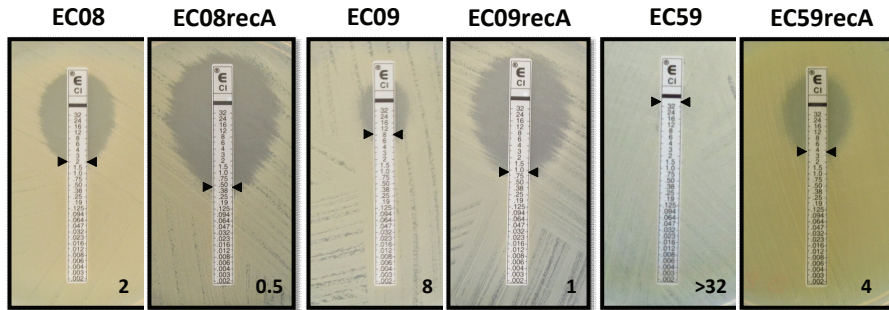

Supplement: FIG S2 [file mbo005173521sf2.pdf]

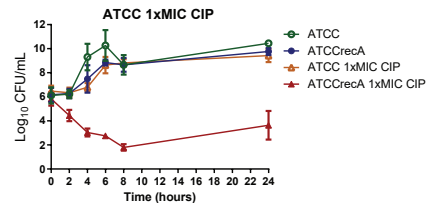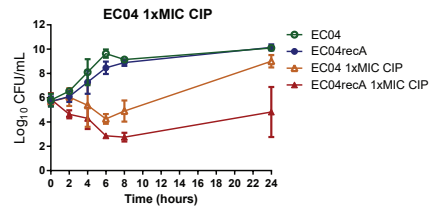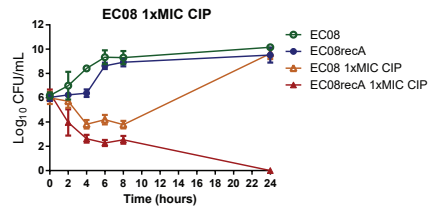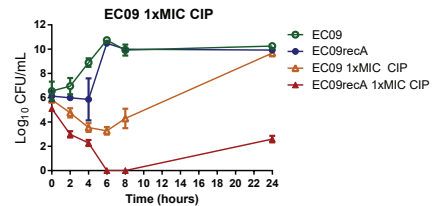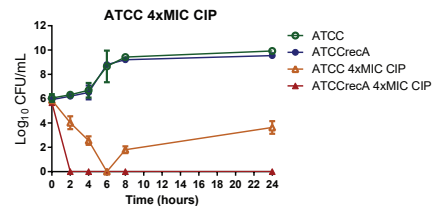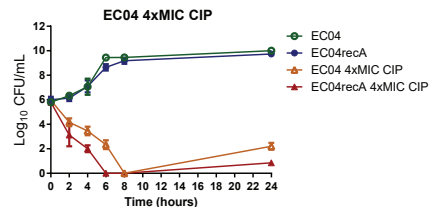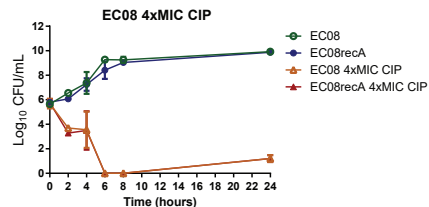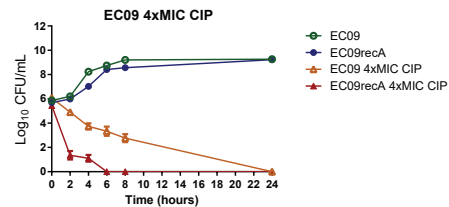

Supplement: FIG S3 [file mbo005173521sf3.pdf]

**A**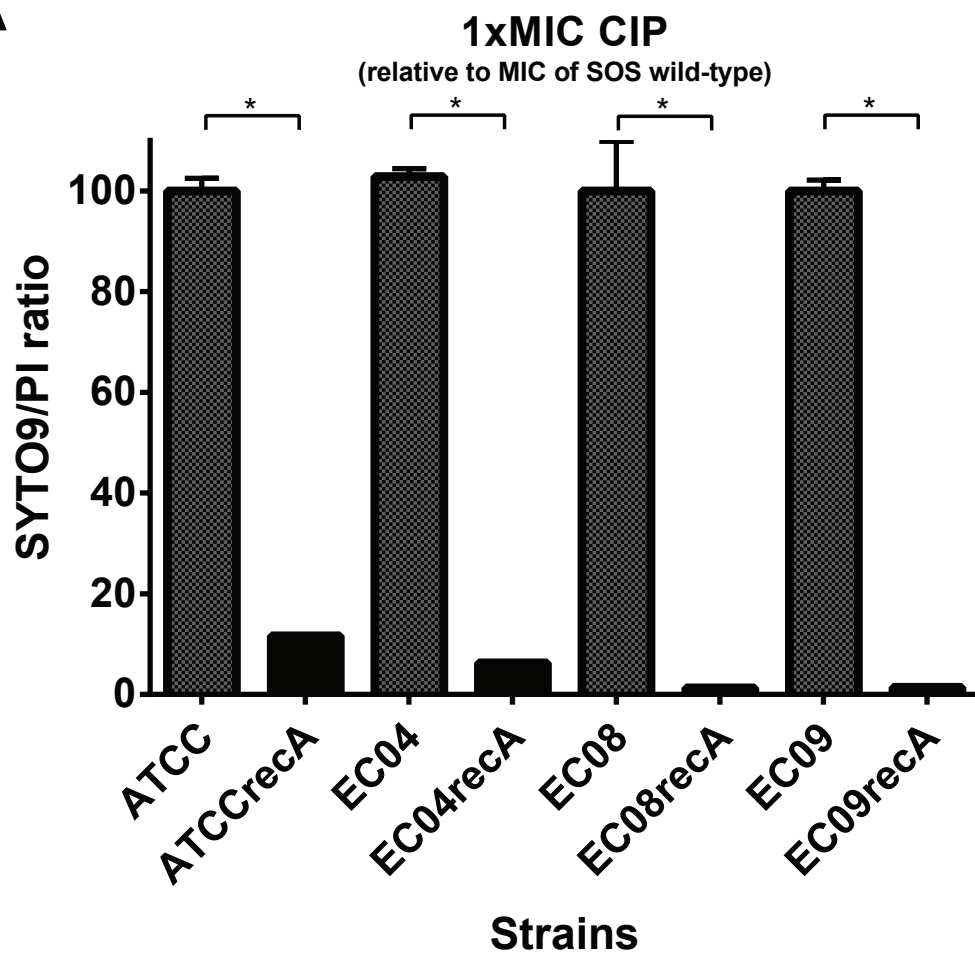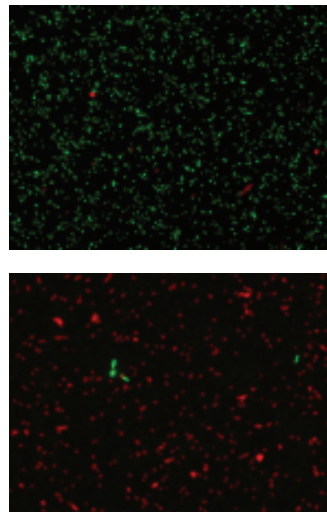**B**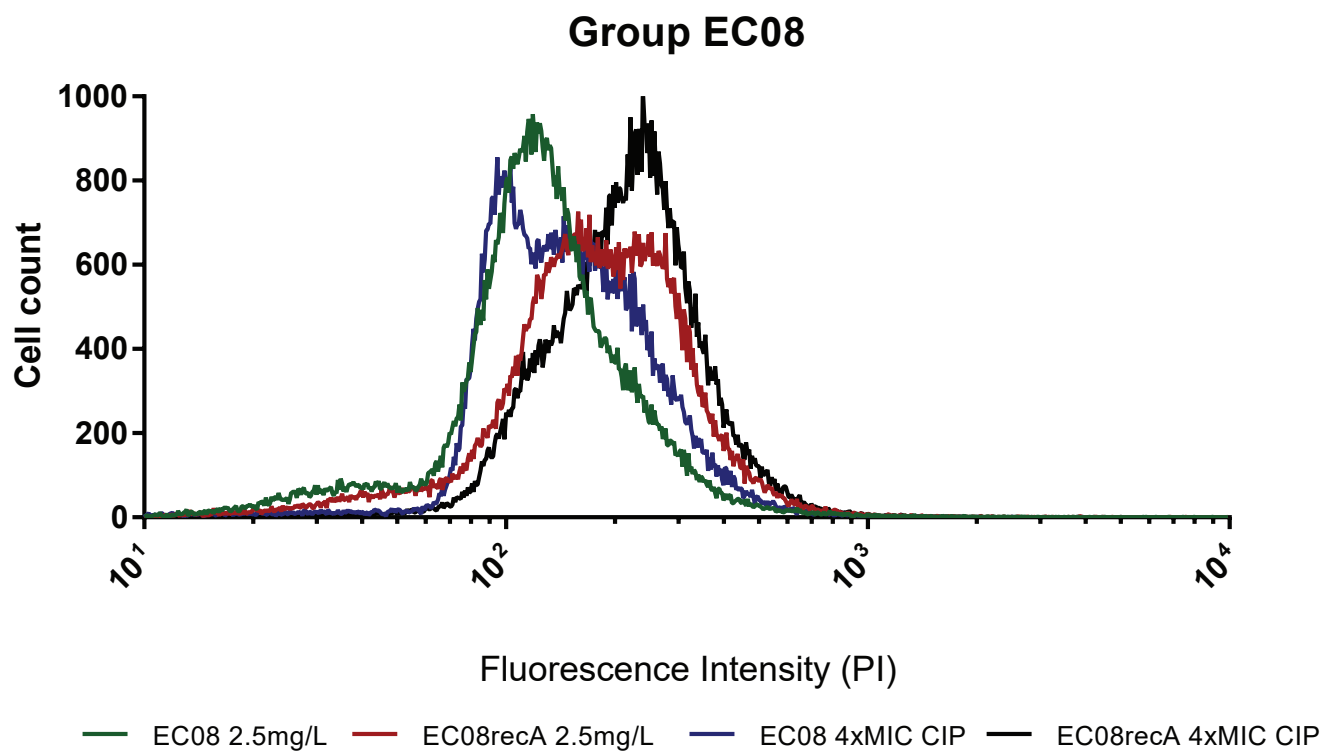

Supplement: FIG S4 [file mbo005173521sf4.pdf]

Figure S5

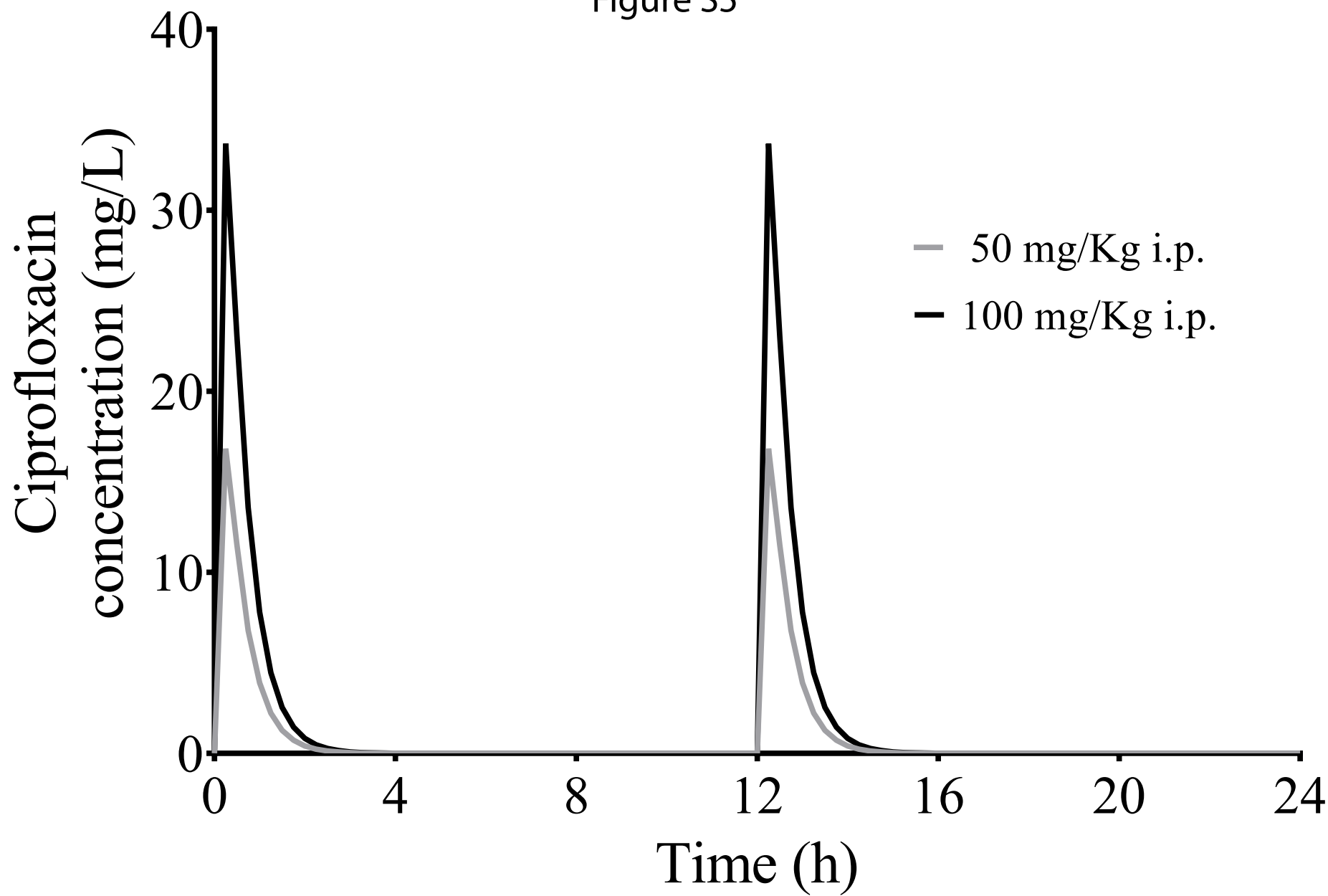

Supplement: FIG S5 [file mbo005173521sf5.pdf]

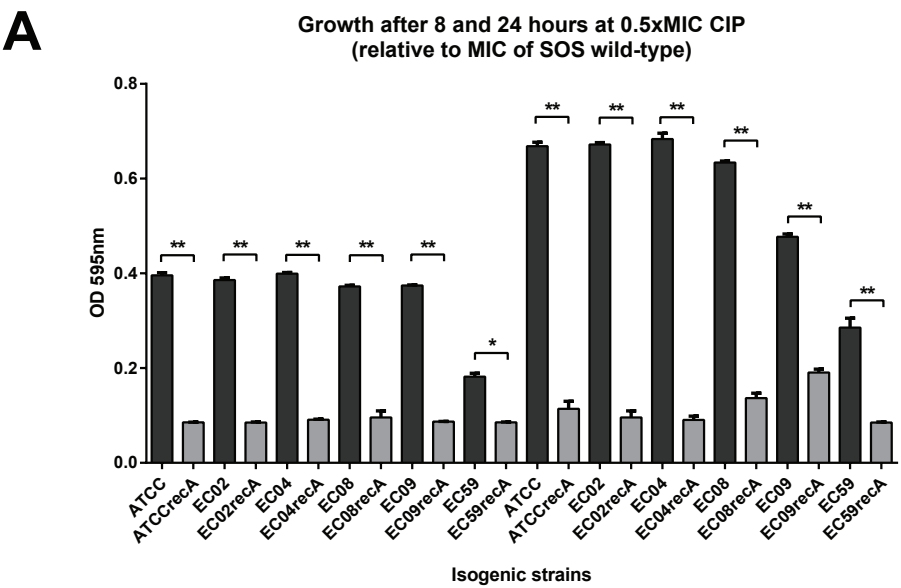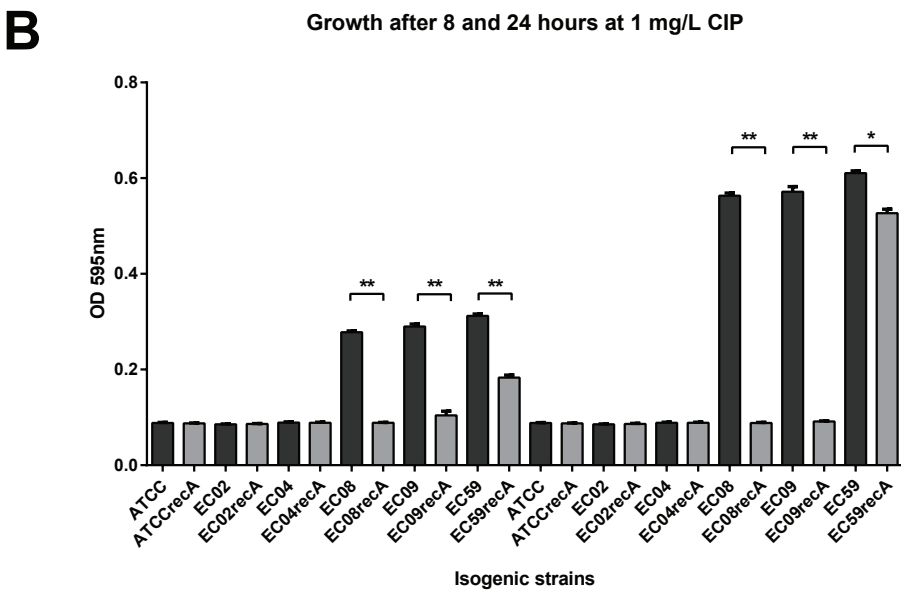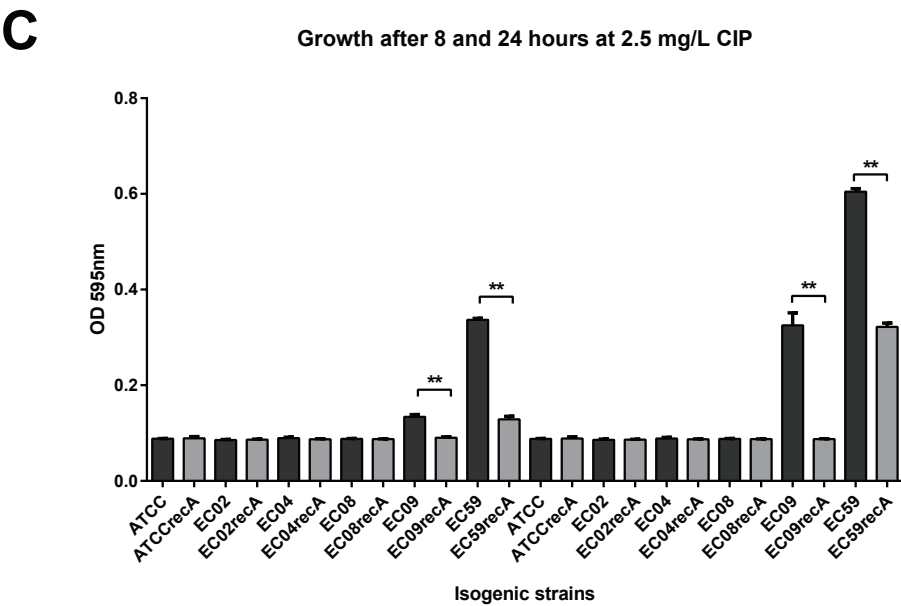

Supplement: FIG S6 [file mbo005173521sf6.pdf]

Figure S7

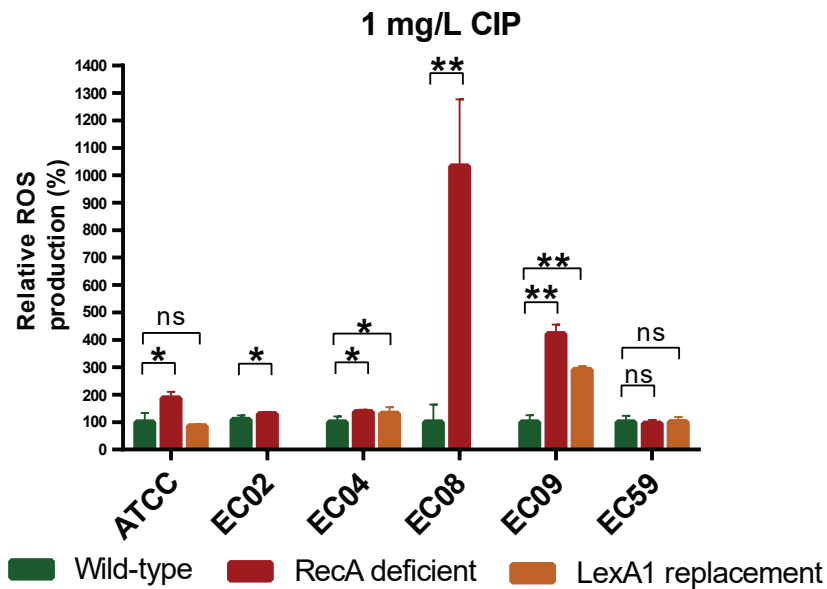

Supplement: FIG S7 [file mbo005173521sf7.pdf]

Figure S8A

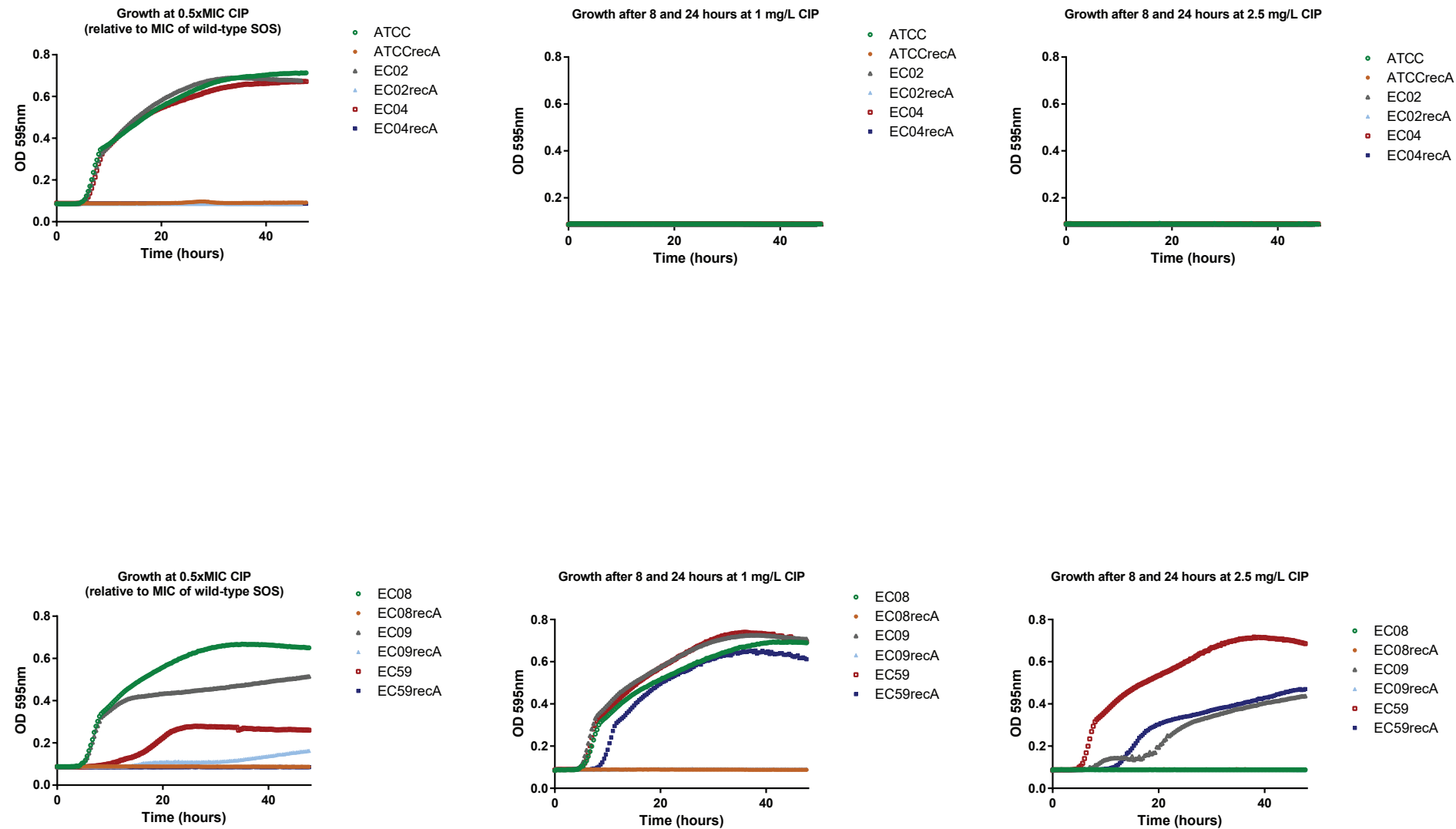

Figure S8B

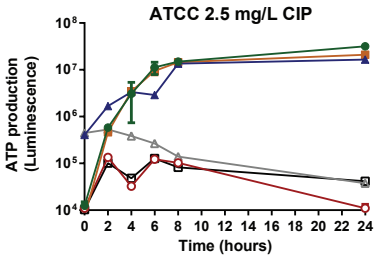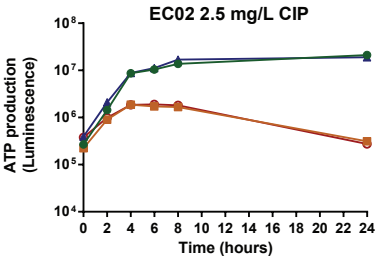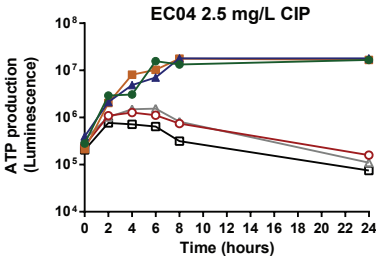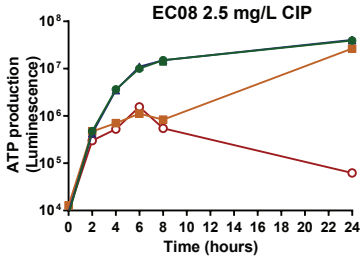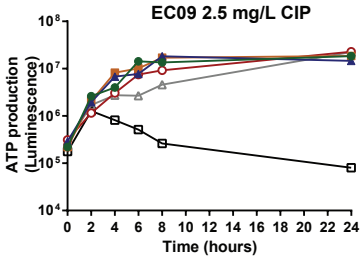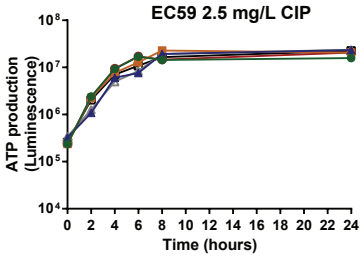

# Figure S8C

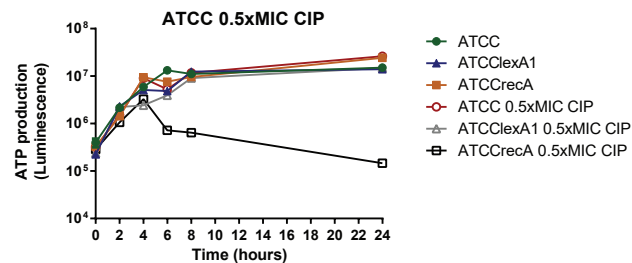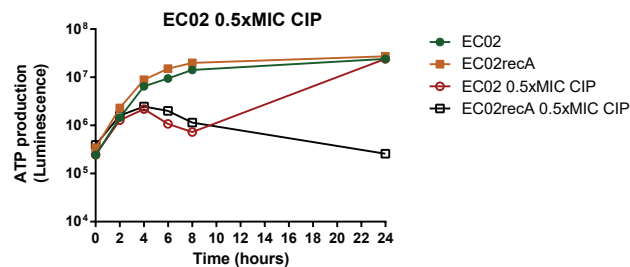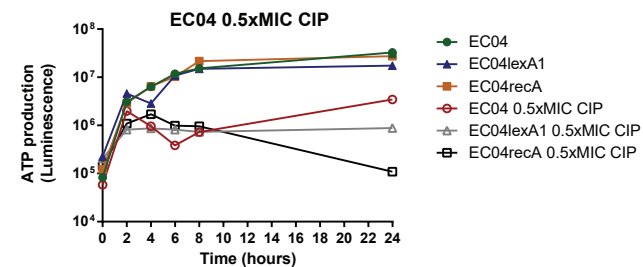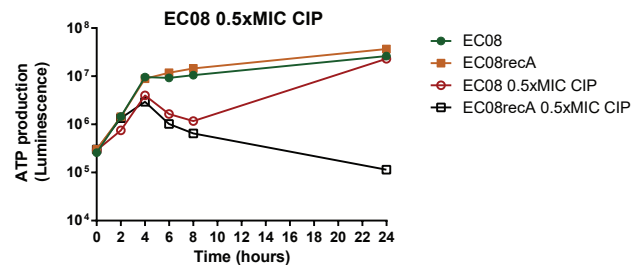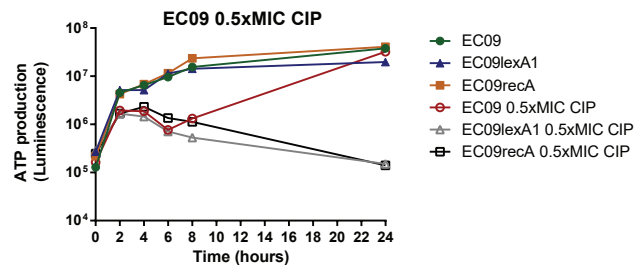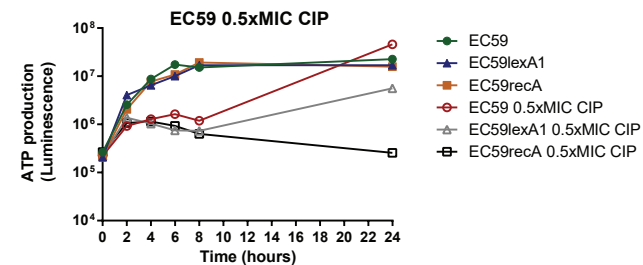

Supplement: FIG S8 [file mbo005173521sf8.pdf]
