# Supplementary material for: Quinolone Resistance Reversion by Targeting the SOS Response
Source: mBio. 2017 Oct 10;8(5):e00971-17. doi: 10.1128/mBio.00971-17 (PMC5635686; doi:10.1128/mBio.00971-17)
Supplement: TEXT S1 [file mbo005173521s1.docx]

**Supplementary Materials and Methods**

**Strains, growth conditions and antimicrobial agents**

Wild-type *E. coli* ATCC 25922 was used as the starting strain for all constructions (Table 1). This strain is routinely used in clinical laboratories as a control for antimicrobial susceptibility tests ([www.atcc.org](http://www.atcc.org)) (1). *E. coli* ATCC 25922 (wild-type) and isogenic EC02, EC04, EC08, EC09 and EC59 strains represent progressive degrees of fluoroquinolone resistance, ranging from susceptible to resistant. These strains harbor multiple chromosomally-mediated (GyrA and/or ParC modifications, a marR deletion that explains acrAB-TolC efflux pump overproduction) and plasmid-mediated (*qnrS*) resistance mechanisms (Table 1). *E. coli* ME12 *lexA1* (ME12 *lexA1* *malB*::Tn9) was used as the source of *lexA* to *lexA1* gene replacement (2).

Liquid or solid Luria Bertani medium (LB) was routinely used. Mueller-Hinton broth (MHB) and M9 minimal medium containing glucose as the sole carbon source were used as required for susceptibility and fluorescence assays, respectively. Strains were grown at 37ºC. 30 mg/L kanamycin (Sigma-Aldrich, Madrid, Spain) in selective media was used for pBK-CMV and pMSrecA-gfp stability (Table S1B).

The following quinolones were used for the different assays: nalidixic acid (Sigma-Aldrich, Madrid, Spain), ciprofloxacin (Sigma-Aldrich), levofloxacin (Sigma-Aldrich), moxifloxacin (Sigma-Aldrich), norfloxacin (Sigma-Aldrich) and ofloxacin (Sigma-Aldrich). Stock solutions were prepared in accordance with Clinical and Laboratory Standards Institute (CLSI) guidelines (www. clsi.org) (1).

**Isogenic strain construction**

*lexA1* mutants (coding for a LexA G80D susbstitution) (2) were obtained by gene replacement, as previously described (Table 1, Table S1B) (3, 4). Briefly, an internal fragment of mutated gene from *E. coli* ME12 *lexA1* strain was cloned into a conventional pBK-CMV vector using specific primers, shown in Table S1B. Then, the mutated fragment was subcloned into the BamH1 site of suicide plasmid pST76-C. The construction was introduced into the corresponding *E. coli* mutant by electroporation. The mutated fragment was inserted into the chromosome by homologous recombination. The resolution process was mediated by the pUC19RP12 vector and confirmed by PCR.

Disruption of the recA gene was carried out with a modified version of the method described by Datsenko and Wanner (5). Briefly, a kanamycin resistance gene from the pKD4 vector was amplified by PCR from genomic DNA using primers with 5′ extensions homologous to the recA gene (see Table S1B). The Red helper plasmid, pKOBEG, a low copy number plasmid vector containing exonuclease and the β and γ functions of phage λ, was used to enhance the recombination process. The kanamycin resistance cassette was then eliminated using helper plasmid, pCP20, which encodes FLP recombinase, and subsequently cured.

The *qnrS* gene (including the promoter sequences ~150bp upstream of the encoding sequence) was cloned into the kanamycin-resistant pBK-CMV vector (in the opposite orientation with respect to the *P*_lac_ promoter) and electroporated into EC59 and its derivative mutants (3).

**Monitoring the induction of the SOS system**

To validate the constructions aimed at inactivating the SOS response, the low-copy plasmid, pMSrecA-gfp, a GFP reporter-promoter fusion, (6) was used to quantitate *recA* promoter activity (SOS induction) after the addition of ciprofloxacin (Table S1B). This construction was introduced into all mutants in our collection by electroporation. After overnight culture in LB, bacterial suspensions were diluted 10^-2^-fold in 5 mL LB (containing kanamycin) for 6 h at 37°C with shaking (250 rpm) in a light-protected environment. Cells were diluted to achieve an OD_625nm_ of 0.1 (*ca.* 10^8^ CFU/mL), then diluted 10^-2^-fold in M9 medium at fixed (1 mg/L or 2.5 mg/L) or sublethal (1xMIC) concentrations of ciprofloxacin, or without ciprofloxacin, and transferred to 96-well, white flat bottom microplates. The microplates were read using the Infinite 200PRO multimode microplate reader (Tecan, Madrid, Spain). The excitation/emission wavelengths used were 485/535 nm. Each experiment was performed at least in quintuplicate. Average fluorescence was monitored for 24 h. Data were normalized to the non-induced control (by extracting the fluorescent background) and relative to bacterial density (OD_595nm_).

**Bacterial growth rates**

After overnight culture in LB at 37ºC, bacterial suspensions were properly diluted to achieve an OD_625nm_ of 0.1 (*ca.* 10^8^ CFU/mL), then diluted 10^-2^-fold in M9 medium and 10^-4^-fold in MHB medium containing sublethal (0.5xMIC) or fixed concentrations of antimicrobial agent (1 mg/L, breakpoint for resistance according to EUCAST, or 2.5 mg/L, human serum Cmax for ciprofloxacin) (7, 8). One hundred and fifty microliters of the diluted bacterial culture were then distributed among 96-well transparent flat bottom plates. The plates were sealed with transparent tape. The cultures were incubated at 37°C on an orbital shaker and agitated (2-mm orbital shaking, 450 rpm, 10 s) for 48 h and measured with an Infinite 200 PRO plate reader (Tecan). Optical density (OD_595nm_) measurements were obtained every 20 min. At least six biological replicas were measured for each condition in at least two independent assays.

**Microbial cell viability assays (ATP production)**

The luminescence-based BacTiter-Glo Microbial Cell Viability Assay (Promega) was used to determine the metabolic activity and viability of all mutants in this collection (Table 1) based on the SOS system induction status. This is a homogeneous procedure that determines the number of viable bacterial cells in a culture by quantifying ATP production (as an indicator of metabolically active cells). Cells were exposed to sublethal (0.5xMIC) ciprofloxacin concentrations of the tested strains harbouring a non-modifed SOS system (*i.e.* intact *recA* and *lexA* genes) or fixed concentrations (1 mg/L meaning breakpoint for resistance according to EUCAST or 2.5 mg/L meaning serum Cmax for ciprofloxacin) in MHB (7, 8). Growth in drug-free broth was evaluated in parallel as a control. Cultures were incubated at 37ºC, with shaking at 250 rpm. An initial inoculum of 10^6^ CFU/mL was used in all experiments. ATP production was determined at 0, 2, 4, 6, 8 and 24 h. At each time-point, the cells were washed with PBS and 100 µL of culture was mixed in a 1:1 proportion with BacTiter-Glo Reagent and distributed among 96-well, white flat bottom plates. ATP concentrations were measured using a luminometer (Veritas, Turner Biosystems). At least six biological replicas under each condition were measured in at least two independent assays.

**Quantification of live/dead bacteria by fluorescence**

The Molecular Probes™ LIVE/DEAD® BacLight™ Bacterial Viability kit (Invitrogen) was used to show the impact of SOS inactivation after a short period of antimicrobial exposure. This two-color fluorescence assay of bacterial viability uses a mixture of the green and red fluorescent nucleic acid stains, SYTO9 and propidium iodide (PI), respectively. With appropriate mixtures of the SYTO9 and PI stains, bacteria with intact cell membranes stain fluorescent green, whereas bacteria with damaged membranes stain fluorescent red, according to the kit instructions.

Cells were exposed at 1xMIC and 4xMIC ciprofloxacin concentration of the tested strains harbouring a non-modifed SOS system (*i.e.* intact *recA* and *lexA* genes) or to a fixed concentration (1 mg/L, the breakpoint for resistance according to EUCAST; or 2.5 mg/L, the serum Cmax for ciprofloxacin) (7, 8).

Fluorescence intensity was measured as follows in an Infinite 200Pro multireader. First, cells were cultured overnight in LB and diluted to achieve an OD_625nm_ of 0.1 (*ca.* 10^8^ CFU/mL), then diluted 10^-2^-fold in MHB and exposed to ciprofloxacin. After 4h, samples were centrifuged at 4,500 rpm for 5 min at 4 °C and washed once in ice cold saline solution before staining, in accordance with the kit instructions (1.5 µL of a 3.34 mM solution of SYTO9 in DMSO and 1.5 µL of a 20 mM solution of PI in DMSO in 1 mL of cells). Two hundred microliter aliquots were then transferred in quintuplicate to 96-well microtiter plates. Fluorescence was measured at excitation/emission wavelengths of 485/535 nm for SYTO9, and 535/590 nm for PI. Data were normalized as a SYTO9/PI ratio.

Fluorescence microscopy assays were performed using the Axio Vert.A1 system (Zeiss) and ZEN (blue edition, 2011) software. ImageJ 1.48v software (National Institute of Health, USA) was used for image analysis. Aliquots (2 mL) of exponentially growing cells (~10^8^ CFU/mL) were incubated with ciprofloxacin and shaken (250rpm) for 4 h at 37°C, as previously indicated, then prepared for staining, as described above. Two microliters of each culture was used to prepare the samples, and images of the cells were obtained using an AxioCam 503 color microscope camera fitted with a 40x objective.

The number of viable cells was also quantified by flow cytometry (CYTOMICS FC500-MPL, Beckman Coulter). For this assay, cells were cultured in the same way and exposed to ciprofloxacin for 60 min. To prepare the cells for measurement, 1 mL of cell culture was washed once in ice cold PBS and resuspended in 1 mL of saline solution, stained according to the kit instructions, then incubated for 15 min before counting. The following PMT voltages were used: FL1 420, FL3 560. At least 10,000 cells per sample were collected. Flow cytometry acquisition was performed at a low flow rate (~30 events/s) (9).

**Mice**

Male immunocompetent C57BL/6 mice were obtained from the University of Seville and allowed to acclimatize for at least 1 week before the experiments began. The mice were housed in individually ventilated cages under specific pathogen-free conditions and water and standard food were given ad libitum. All animals were cared for in accordance with national guidelines and the studies were performed in accordance with guidelines approved by the Ethics and Clinical Research Committee of the Virgen Macarena and Virgen del Rocio University Hospitals (Reference Number 1086-N-15).

**Pharmacokinetics and pharmacodynamics**

Pharmacokinetic serum data from our previous work was used to construct a mathematical pharmacokinetic model (10). The observed ciprofloxacin concentrations in mice were fitted to a two-compartment model (intraperitoneal space and blood) using ADAPT5 software using the following differential equations (11).

$$\boldsymbol{XP}_{\boldsymbol{1}}\boldsymbol{=}\boldsymbol{R}_{\boldsymbol{1}}\boldsymbol{-}\left( \boldsymbol{X}_{\boldsymbol{1}}\boldsymbol{\times}\boldsymbol{K}_{\boldsymbol{cp}} \right)\boldsymbol{+(}\boldsymbol{X}_{\boldsymbol{2}}\boldsymbol{\times}\boldsymbol{K}_{\boldsymbol{pc}}\boldsymbol{)}$$

$$\boldsymbol{XP}_{\boldsymbol{2}}\boldsymbol{=}\left( \boldsymbol{X}_{\boldsymbol{1}}\boldsymbol{\times}\boldsymbol{K}_{\boldsymbol{cp}} \right)\boldsymbol{-(}\boldsymbol{X}_{\boldsymbol{2}}\boldsymbol{\times}\boldsymbol{K}_{\boldsymbol{pc}}\boldsymbol{)-(}\boldsymbol{X}_{\boldsymbol{2}}\boldsymbol{\times}\frac{\boldsymbol{Cl}}{\boldsymbol{V}_{\boldsymbol{p}}}\boldsymbol{)}$$

Where R_1_ is the infusion of ciprofloxacin into the central compartment (peritoneum). X_1_ and X_2_ are the amounts of ciprofloxacin (mg) in the central compartment and peripheral compartment (blood), respectively. Cl is the clearance of ciprofloxacin from the peripheral compartment (L/h). V_p_ (L) is the volume of distribution of the drug in the peripheral compartment, and K_cp_ and K_pc_ are the first-order intercompartmental rate constants.

A range of dosages were simulated in order to obtain a favorable pharmacokinetic parameter of AUC_0-24h_/MIC ~50 or ~100, adjusted to the SOS-deficient strain in the isogenic pair EC08/EC08recA (EC08recA, ciprofloxacin MIC=0.5 mg/L).

**Experimental model**

Male immunocompetent C57BL/6 mice weighing 16 to 18 g were used. Using a murine model of peritoneal sepsis, the minimum lethal dose (MLD) for EC08 and EC08recA strains was determined as follows. Briefly, groups of 10 mice were infected intraperitoneally with 0.5 mL of bacterial suspension over a range of concentrations. Mortality was then monitored for 72h. The MLD for EC08 and EC08recA strains was set as the starting inoculum for further experiments (0.5 ml in a 1:10 dilution of an overnight culture, about 5x10^7^ CFU/mL). Next, the murine model of peritoneal sepsis was used to evaluate the efficacy of ciprofloxacin between EC08 and EC08recA. Mice were infected intraperitoneally using the MLD. Two hours post-infection, antimicrobial therapy started. Animals were randomly assigned to different therapeutic groups: Group 1: ciprofloxacin administered intraperitoneally at 50 mg/Kg q12h; Group 2: ciprofloxacin administered intraperitoneally at 100 mg/Kg q12h; and the control group (no treatment).

At 24 h, the bacterial loads in the spleens of 15 mice per strain and ciprofloxacin dosage were determined. The spleens of mice that died during the experiment or were sacrificed (by lethal dose of sodium thiopental) were aseptically extracted, then weighed and processed for quantitative cultures, after being homogenized in 2 mL of sterile saline solution (Stomacher 80; Tekmar Co., Cincinnati, OH, USA). After a 10-fold dilution, aliquots of 100 µL were plated on Mueller-Hinton agar plates and incubated at 37ºC overnight.

**ROS detection using DCFH-DA**

In order to determine whether changes in susceptibility, associated with an active or inactive SOS response, correlated with ROS formation, the radical-senstitive reporter dye, fluorescent 2’,7’–dichlorofluorescin diacetate (DCFH-DA) (Sigma-Aldrich), was used to examine the levels of oxidizing radicals. Overnight cultures were diluted 10^-2^-fold in M9 minimal medium and grown to an OD_600nm_ of ~0.2 before adding the antibiotic. DCFH-DA was added after 2.5 h at a final concentration of 5 µM. Microplate assays were read using an Infinite 200 PRO multimode microplate reader (Tecan). Excitation/emission wavelengths used were 485/535 nm. Average fluorescence was determined at 0 h (baseline) and at 18 h for microplate assays. Data were normalized to a no-dye control (background fluorescence) and OD_595nm_ (bacterial density). Bacterial cells were treated with ciprofloxacin at a concentration of 1 mg/L. At least six biological replicas per condition were measured in at least two independent assays.

**Supplementary Results**

**Generation of recA-deficient and lexA mutant strains in E. coli**

While other studies have used strains with inactivated LexA or deleted recA in highly-susceptible wild-type phenotypes to validate the SOS pathway as a therapeutic target, none of them have evaluated the spectrum of SOS activity against resistant or low-level resistant phenotypes in order to bring about a process of re-sensitization that could be used as an additional strategy for therapeutic use against resistant bacteria. To this end, we derived a series of E. coli ATCC 25922 strains that enabled us to systematically examine the consequences of altered SOS activity in terms of re-sensitization using a model against fluoroquinolones. The aim was to engineer strains with lexA variations (*lexA1*) or deleted recA with respect to the wild-type SOS phenotypes that would span the range of natural SOS system induction, partial SOS activation and finally constitutive SOS inactivation in a set of multiple quinolone resistance genotypes and phenotypes ([Figure](http://msphere.asm.org/content/1/4/e00163-16#F1) 1, Table 1).

The LexA1 enzyme that was generated in this set of strains with the gene replacement technique was a “hypoactive” G80D mutant, which has a very low cleavage rate (12); deletion of the *recA* gene (the major SOS activator and sensor of DNA damage) led to constitutive SOS inactivation.

Several approaches were taken to verify the effectiveness of SOS inactivation in our collection. Induction of the SOS system was monitored with pMSrecA-gfp, the gfp reporter-promoter fusion (6) to quantify *recA* promoter activity (SOS induction) after the addition of ciprofloxacin at sublethal (1xMIC with respect or not to the MIC of the SOS wild-type) and fixed concentrations (of 1 mg/L or 2.5 mg/L) (data at 4 h are shown) (Figure S1). Both *recA*-deleted variants and *lexA*1 mutants showed that induction of the SOS response (pMSrecA-gfp) was drastically reduced under these conditions. For example, at 1xMIC of ciprofloxacin, the reduction was of 90-94% in Δ*recA* variants and 80-88% in *lexA*1 mutants. At the fixed 1 mg/L concentration of ciprofloxacin, the reduction was in the range of 92-97% in Δ*recA* variants and around 86% in *lexA*1 mutants, and at 2.5 mg/L, it was around 98% in Δ*recA* variants and around 90% in *lexA*1 mutants. (Figure S1) It was not possible to evaluate the results for the ATCC wild-type strain at the fixed ciprofloxacin concentrations due to its high level of susceptibility, which explains the elevated level of DNA damage and cell death. Interestingly, even the EC02 strain (coding only for a S83L substitution and with a low MIC value, 0.06 mg/L) had a detectable SOS response under the relevant therapeutic concentrations.

In addition, a comparison of isogenic pairs according to SOS system induction status using disk diffusion susceptibility tests and/or fluorescent microscopy showed these differences of SOS induction in the generated collection (Figure S1).

**Monitoring bacterial *in vitro* growth in the presence of quinolones**

*In vitro* growth curves were analyzed at subinhibitory concentrations of ciprofloxacin (0.5xMIC based on the susceptibility of strains with unmodified SOS systems) or at fixed concentrations (1 mg/L breakpoint for resistance according to EUCAST, or 2.5 mg/L serum Cmax for ciprofloxacin) (7, 8). At fixed concentrations, growth was observed only in the specific cases of the isogenic pairs EC08/EC08recA, EC09/EC09recA and EC59/EC59recA. At 1 mg/L of ciprofloxacin, only strains EC08 (and not EC08recA), EC09 (and not EC09recA), EC59 and EC59recA grew (Figure S6) at both 8 h and 24 h (this behavior was stable after 48 h). Growth rates (OD_595nm_) for EC08/EC08recA were 0.27/0.08 at 8 h and 0.56/0.08 at 24 h (P<0.001). Interestingly, EC59recA clearly showed delayed growth (Figure S6, Figure S8A). Growth curve studies with 2.5 mg/L of ciprofloxacin showed that only the EC09 (and not EC09recA), EC59 and EC59recA strains grew (Figure S6) at both 8 h and 24 h (this behavior was stable after 48 h). Growth rates (OD_595nm_) for EC09/EC09recA were 0.13/0.08 at 8 h and 0.32/0.08 at 24 h; for EC59/EC59recA, they were 0.33/0.12 at 8 h and 0.61/0.32 at 24 h (P<0.001). In the case of EC59recA, delayed growth was even more obvious (P<0.001). In the presence of subinhibitory concentrations of ciprofloxacin (0.5xMIC according to the susceptibility of strains with unmodified SOS systems), obvious differences were also observed for all isogenic pairs and growth was observed only in strains harboring the wild-type SOS response (OD_595nm_: ~0.4 vs ~0.08 at 8 hours, P<0.001) (Figure S6, Figure S8A). Worthy of note, at subinhibitory concentrations, the most resistant strains, EC08, EC09 and EC59, showed delayed growth after 24 h of incubation, compared with the most susceptible strains, ATCC, EC02 and EC04. These data support the efficacy of inactivating the SOS response in LLQR or resistant strains in order to prevent bacterial growth at relevant fluoroquinolone concentrations, or in accordance with MIC values, over shorter and longer periods of time. Moreover, a clear correlation were observed when bacterial viability was measured in terms of ATP production (Figure S8BC).

**SOS inactivation leads to increased ROS in resistant strains**

At the test concentration (1 mg/L of ciprofloxacin), only minor but significant differences of ROS production were observed for susceptible isogenic groups (ATCC, EC02, EC04) (p<0.05). No significant differences were observed in the highly resistant group (EC59). The tested concentration was too high in the first case and too low in the second case to show clearer differences in ROS production. However, at this clinically relevant concentration of ciprofloxacin, strong significant differences (p<0.001) were observed in the isogenic EC08 and EC09 groups, the ones in which a process of resensitization of clinical category had previously been noted. SOS inactivation led to 2.9-fold and 4.2-fold increases in ROS production, associated with LexA1 modification and *recA* gene deletion, compared with the EC09 mutant, and to a 10.3-fold increase, associated with a *recA* gene deletion, compared with the EC08 mutant (Figure S7). No differences were observed in the absence of the drug, indicating that the observed differences were related to drug response and SOS modulation (data not shown). This association between increased ROS production and SOS inactivation following treatment with a bactericidal antibiotic (1 mg/L of ciprofloxacin) suggests that ROS may actively contribute to the bactericidal activity of fluoroquinolones.

**Supplementary References**

1. Clinical and Laboratory Standards Institute. Performance Standards for Antimicrobial Susceptibility Testing: Twenty-sixth Informational Supplement M100-S26. CLSI, Wayne, PA, USA, 2016.

2. Elez M, Radman M, Matic I. 2007. The frequency and structure of recombinant products is determined by the cellular level of MutL. Proc Natl Acad Sci USA 104:8935–8940.

3. Machuca J, Briales A, Labrador G, Diaz-de-Alba P, Lopez-Rojas R, Docobo-Perez F, Martinez-Martinez L, Rodriguez-Bano J, Pachon ME, Pascual A, Rodriguez-Martinez JM. 2014. Interplay between plasmid-mediated and chromosomal-mediated fluoroquinolone resistance and bacterial fitness in *Escherichia coli*. J Antimicrob Chemother 69:3203–3215.

4. Posfai G, Kolisnychenko V, Bereczki Z, Blattner FR. 1999. Markerless gene replacement in *Escherichia coli* stimulated by a double-strand break in the chromosome. Nucleic Acids Res 27:4409–4415.

5. Datsenko KA, Wanner BL. 2000. One-step inactivation of chromosomal genes in *Escherichia coli* K-12 using PCR products. Proc Natl Acad Sci USA 97:6640–6645.

6. Zaslaver A, Bren A, Ronen M, Itzkovitz S, Kikoin I, Shavit S, Liebermeister W, Surette MG, Alon U. 2006. A comprehensive library of fluorescent transcriptional reporters for *Escherichia coli*. Nat Methods 3:623–628.

7. EUCAST. Clinical Breakpoints and Epidemiological Cut-off Values. 2016. http://www.eucast.org/clinical_breakpoints/.

8. Bennet JE, Dolin R, Blaser MJ. Mandell, Douglas, and Bennett’s Principles and Practice of Infectious Diseases. Elsevier Saunders, 2015; p427.

9. Kohanski MA, Dwyer DJ, Collins JJ. 2010. How antibiotics kill bacteria: from targets to networks. Nat Rev 8:423–435.

10. Domínguez-Herrera J, Velasco C, Docobo-Pérez F, Rodríguez-Martínez JM, López-Rojas R, Briales A, Pichardo C, Díaz-de-Alba P, Rodríguez-Baño J, Pascual A, Pachón J. 2013. Impact of *qnrA1*, *qnrB1* and *qnrS1* on the efficacy of ciprofloxacin and levofloxacin in an experimental pneumonia model caused by *Escherichia coli* with or without the GyrA mutation Ser83Leu. J Antimicrob Chemother 68:1609–1615.

11. D’Argenio, D.Z., A. Schumitzky and X. Wang. ADAPT 5 User’s Guide: Pharmacokinetic/Pharmacodynamic Systems Analysis Software. Biomedical Simulations Resource, Los Angeles, 2009.

12. Mo CY, Birdwell LD, Kohli RM. 2014. Specificity determinants for autoproteolysis of LexA, a key regulator of bacterial SOS mutagenesis. Biochemistry 53:3158–68.
